# Consensus Pathways Implicated in Prognosis of Colorectal Cancer Identified Through Systematic Enrichment Analysis of Gene Expression Profiling Studies

**DOI:** 10.1371/journal.pone.0018867

**Published:** 2011-04-25

**Authors:** Jesús Lascorz, Bowang Chen, Kari Hemminki, Asta Försti

**Affiliations:** 1 Division of Molecular Genetic Epidemiology, German Cancer Research Center (DKFZ), Heidelberg, Germany; 2 Center for Primary Health Care Research, Clinical Research Center, Lund University, Malmö, Sweden; Baylor College of Medicine, United States of America

## Abstract

**Background:**

A large number of gene expression profiling (GEP) studies on prognosis of colorectal cancer (CRC) has been performed, but no reliable gene signature for prediction of CRC prognosis has been found. Bioinformatic enrichment tools are a powerful approach to identify biological processes in high-throughput data analysis.

**Principal Findings:**

We have for the first time collected the results from the 23 so far published independent GEP studies on CRC prognosis. In these 23 studies, 1475 unique, mapped genes were identified, from which 124 (8.4%) were reported in at least two studies, with 54 of them showing consisting direction in expression change between the single studies. Using these data, we attempted to overcome the lack of reproducibility observed in the genes reported in individual GEP studies by carrying out a pathway-based enrichment analysis. We used up to ten tools for overrepresentation analysis of Gene Ontology (GO) categories or Kyoto Encyclopedia of Genes and Genomes (KEGG) pathways in each of the three gene lists (1475, 124 and 54 genes). This strategy, based on testing multiple tools, allowed us to identify the oxidative phosphorylation chain and the extracellular matrix receptor interaction categories, as well as a general category related to cell proliferation and apoptosis, as the only significantly and consistently overrepresented pathways in the three gene lists, which were reported by several enrichment tools.

**Conclusions:**

Our pathway-based enrichment analysis of 23 independent gene expression profiling studies on prognosis of CRC identified significantly and consistently overrepresented prognostic categories for CRC. These overrepresented categories have been functionally clearly related with cancer progression, and deserve further investigation.

## Introduction

Colorectal cancer (CRC) is the third most common cancer and the fourth-leading cause of cancer death worldwide, with a lifetime risk in Western European and North American populations around 5% [1].

Many gene expression profiling (GEP) studies on CRC have been performed in the last decade using microarray technology. According to their potential clinical applications, they can be classified into three groups [2]: studies on carcinogenesis process, studies on prognosis prediction, and studies on treatment response prediction. They show little overlap in the identified genes, and no reliable signature useful in clinical practice has been found. Currently, the International Union Against Cancer (UICC) TNM classification of malignant tumours based on clinicopathological staging remains the standard for CRC prognostication [3].

We focused on the studies on prognosis prediction, which comprise a heterogeneous group of GEP studies. They aim to identify a gene expression profile to discriminate more aggressive from less aggressive CRC, based on different features related to disease progression, such as the existence of recurrence, the presence of metastasis, or survival data. To date, only one meta-analysis of ten GEP studies has reported a list of 13 genes differentially expressed in CRC with good versus bad prognosis, reported by at least two independent studies [4].

Multiple reasons have been proposed to explain this lack of reproducibility in the GEP studies on CRC, such as underpowered studies, lack of validation of results, differences in experimental protocol and statistical pitfalls in analysing microarray expression data for cancer outcome [3]. Changes in biological characteristics require coordinated variation in expression of gene sets which regulate biological activity, and this information can hardly be extracted from changes in expression of individual genes when overlapping among studies is so low [5]. Enrichment analysis tools, which estimate overrepresentation of particular gene categories or pathways in a gene list, are a promising strategy to identify biological categories implicated in the investigated process [6].

A comprehensive analysis of available bioinformatic enrichment tools has recently been published [6]. Based on the algorithm applied, the enrichment tools can be classified into three classes: singular enrichment analysis (SEA or class I); gene set enrichment analysis (GSEA or class II); and modular enrichment analysis (MEA or class III). In all tools, the input list of genes is mapped to the biological terms in databases, and then statistical analysis examines the enrichment of gene members for each of the annotation terms and corrects for multiple testing [6]. We applied several SEA tools for the same input gene lists, and only enriched categories obtained with several tools were considered indicative of genuine prediction. This strategy, based on testing multiple tools, is recommended in order to obtain the most satisfactory results [7].

Gene Ontology (GO) [8] and Kyoto Encyclopedia of Genes and Genomes (KEGG) [9] are the two main annotation databases collecting biological knowledge of genes, which make them very suitable for bioinformatics scanning for enrichment analysis [6]. Currently, GO contains information for 18261 human gene products, while KEGG maps 373 different pathways. Our goal was to identify the functional categories (GO terms and KEGG pathways) that are consistently overrepresented in a statistically significant way in the list of differentially expressed genes inferred from the GEP studies on CRC prognosis. We first collected data from the 23 published independent GEP studies on prognosis of CRC to extract the genes reported in at least two of them, and then these genes were used for the systematic enrichment analysis with several independent SEA tools. This way, we overcame the lack of reproducibility observed in both the genes reported in individual GEP studies and the overrepresented categories reported by enrichment analysis tools, and could identify consistently enriched categories.

## Results

### Meta-analysis of the GEP studies

A total of 1897 different gene identifiers (IDs) were reported to be differentially expressed in the 23 independent GEP studies on prognosis of CRC (Table 1). From them, the number of unique, mapped genes was 1475, of which 603 genes were up-regulated and 794 down-regulated in poor prognosis samples, while 78 had an opposite direction in expression change between single studies. From the 1475 genes, 124 genes (8.4%) were reported in more than one GEP study (115 in two, and nine in three studies), 19 of them (15.3%) were up-regulated in poor prognosis samples in two studies, 35 down-regulated (28.2%), and 70 with contrasting direction in expression change between two studies. Thus, 54 out of the 124 genes (43.5%) reported the same direction in gene expression change in two different GEP studies. From the nine genes reported in three studies (ATP5C1, CA2, CYP51A1, FN1, HSP90AB1, IQGAP1, RPS5, SPP1, and TXN), only CYP51A1 and SPP1 showed the same direction in expression change in all three studies (Table S1). All these nine genes were included in the 54 gene list. There was no tendency of the genes reported by two studies to come up more frequently from two GEP studies investigating the same feature related to disease prognosis (existence of recurrence, presence of metastasis or survival) than from any two studies. The seven studies investigating recurrence reported 541 unique genes, 15 of them (2.8%) in two studies. The 13 studies related to metastasis reported 934 unique genes, with 50 of them (5.3%) in two studies. Finally, the two studies related to survival reported 34 unique genes, none of them common for both studies.

**Table 1:** Gene expression profiling studies on CRC prognosis included in the present study.

First author	Ref.	Year	Platform	Samples	Study design	Reported gene identifiers (IDs)	Unique, mapped genes*
Agrawal	[35]	2002	Affymetrix U95A	60 p.t.	A	107	96
Arango	[13]	2005	Affymetrix U133A	25 p.t.	B	234	220
Bandres	[36]	2007	Oligo array	16 p.t.	A	8	6
Barrier (1)	[28]	2005	Affymetrix U133A	12 p.t.+a.m.	B	47	34
Barrier (2)	[30]	2005	Affymetrix U133A	25 p.t.+a.m.	B	100	94
Barrier (3)	[29]	2006	Affymetrix U133A	50 p.t.	B	30	20
Barrier (4)	[31]	2007	Affymetrix U133A	24 p.t.+a.m.	B	70	63
Bertucci	[14]	2004	cDNA array	22 p.t.+a.m.	A	290	234
Cavalieri	[37]	2007	Agilent 1A	19 p.t.	C	8	8
D'Arrigo	[38]	2005	cDNA array	20 p.t.	A	29	19
Eschrich	[39]	2005	cDNA array	78 p.t.	C	43	26
Fritzmann	[15]	2009	Affymetrix U95A	41 p.t., 25 m.	D	121	115
Garman	[40]	2008	Affym. U95A/133A	52 p.t.	B	50	45
Grade	[41]	2007	Oligo array	73 p.t.	A	68	66
Jiang	[32]	2008	Affymetrix U133A	123 p.t.	B	7	7
Jorissen	[16]	2009	Affym. U133Plus	293 p.t.	D	128	116
Ki	[42]	2007	cDNA array	23 p.t.+m.	D	46	43
Kleivi	[43]	2007	Agilent 1A	18 p.t., 4 m.	D	40	40
Komuro	[44]	2005	cDNA array	89 p.t.	A	62	60
Kwon	[45]	2004	cDNA array	12 p.t.	A	60	53
Liersch	[46]	2009	cDNA array	30 p.t.	B	20	15
Lin	[47]	2007	Oligo array+Affym.	204 p.t.	B	35	32
Smith	[48]	2009	Affym. U133Plus	55 p.t.	E	34	34
Wang	[33]	2004	Affymetrix U133A	74 p.t.	B	23	20
Watanabe (2)	[49]	2009	Affym. U133Plus	36 p.t.	B	45	30
Watanabe (1)	[50]	2009	Affymetrix U133A	89 p.t.	A	73	57
Yamasaki	[51]	2007	cDNA array	32 p.t., 32 m.	D	119	82

*Number of unique, annotated mapped genes obtained by converting the originally reported gene identifiers (IDs) in each single study to the official HUGO gene symbol. p.t., primary tumours; a.m., adjacent mucosa; m., metastasis; A, metastasis yes/no; B, recurrence yes/no; C, survival; D, metastasis vs. primary tumours; E, prognosis good/bad.

See Table S1 and Table S2 for a complete list of genes.

### Enrichment analyses

Three gene lists were used for the enrichment analyses: all 1475 genes reported in the 23 independent GEP studies, the 124 genes reported in at least two GEP studies (independently of consistence in expression change between studies), and the 54 genes reported in at least two GEP studies with consistent direction in gene expression change between samples with poor and good prognosis. Ten enrichment tools were used to obtain significantly overrepresented GO Biological Process, GO Molecular Function categories, and KEGG pathways (Tables S3, S4, S5).

The number of reported enriched categories showed a considerable variability among the different tools used (Table 2), although the same significance threshold (P value<0.05 after correction for multiple testing) and analysis conditions (whole genome as reference background and at least two genes from the input list in the enriched category) were applied in all analyses. The resulted P values for enrichment of a single GO or KEGG term often ranged several orders of magnitude between the different tools (Tables S3, S4, S5). In general, the tools GeneCodis [10] and WebGestalt [11] reported more enriched categories than the other tools, and many of the enriched categories were reported only by GeneCodis (Tables S3, S4, S5). GeneCodis also classified a significantly lower number of genes from the input list in the reported enriched GO categories. On the other hand, the GATHER tool [12] reported less enriched categories than the other tools (Table 2).

**Table 2:** Number of overrepresented GO and KEGG categories in the three gene lists for each of the tools used.

	54 gene list			124 gene list			1475 gene list		
Tool name	BP	MF	KEGG	BP	MF	KEGG	BP	MF	KEGG
ConsensusPathDB	n.a.	n.a.	2	n.a.	n.a.	2	n.a.	n.a.	1
DAVID	0	0	0	0	1	0	95	13	5
FatiGO	1	0	n.a.	0	6	n.a.	53	4	n.a.
GATHER	0	n.a.	0	1	n.a.	0	11	n.a.	1
GeneCodis	26	17	8	54	35	21	115	80	116
GOTM	10	10	n.a.	10	10	n.a.	10	10	n.a.
g:Profiler	9	0	0	16	1	1	181	18	4
Ingenuity*	77		0	69		1	61		159
ToppFun	35	3	0	29	17	1	234	34	10
WebGestalt	40	12	13	40	25	34	40	40	136

Only categories significantly associated after correction for multiple testing (P value<0.05) is shown. BP, Gene Ontology Biological Process; MF, Gene Ontology Molecular Function; KEGG, Kyoto Encyclopedia of Genes and Genomes. n.a., database not applicable.

*Results of the enrichment analysis using the Ingenuity software have to be considered separately, since the software makes use of its own databases, Top Bio Function and Top Canonical Pathways.

### Identification of consistently enriched categories

**Figure 1:**
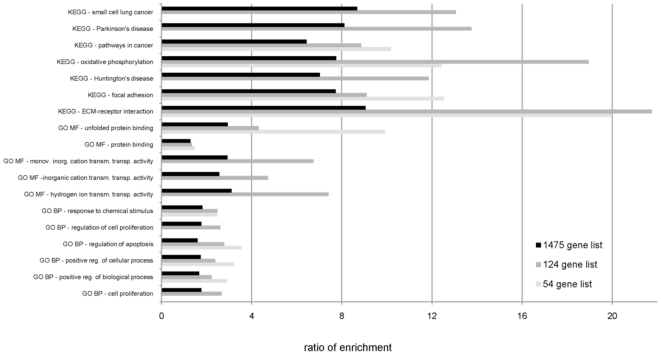
Bar chart of enrichment ratios for GO and KEGG categories in the three gene lists (54, 124, and 1475 genes). Ratio of enrichment = number of observed divided by the number of expected genes from each GO or KEGG category in the gene list (according to WebGestalt or, alternatively, DAVID or GOTM tools). GO BP, Gene Ontology Biological Process; GO MF, Gene Ontology Molecular Function; KEGG, Kyoto Encyclopedia of Genes and Genomes.

Despite the variation in the number of overrepresented categories reported by the different enrichment tools, several categories were reported by many of the tools used. To avoid false positives, we applied two stringent selection thresholds before we considered a category as consistently enriched. First, only the categories reported to be enriched by several tools in a gene list were selected (Table S6). From them, only the categories common in at least two of the three gene lists were considered to be consistently enriched. Using these two selection criteria, six general GO Biological Process categories (cell proliferation, positive regulation of biological process, positive regulation of cellular process, regulation of apoptosis, regulation of cell proliferation, and response to chemical stimulus), five GO Molecular Function categories (hydrogen ion transmembrane transporter activity, inorganic cation transmembrane transporter activity, monovalent inorganic cation transmembrane transporter activity, protein binding, and unfolded protein binding), and seven KEGG pathways (extracellular matrix receptor interaction, focal adhesion, Huntington's disease, oxidative phosphorylation, pathways in cancer, Parkinson's disease, and small cell lung cancer) were consistently overrepresented in the GEP studies on prognosis of CRC (Table 3). The proportion of up- and down-regulated genes was similar within each of the consistently enriched GO and KEGG categories, as in the 124 gene list (data not shown). The ratio of enrichment was higher for the more specific and well-defined KEGG pathways than for the broad GO categories (Figure 1). A high overlap of the individual genes between these 18 categories was also observed (Table 4). Based on this overlap, three biologically meaningful individual category groups were finally obtained:

- A large group including the six general GO Biological Process categories (cell proliferation, positive regulation of biological process, positive regulation of cellular process, regulation of apoptosis, regulation of cell proliferation, and response to chemical stimulus), together with the two GO Molecular Function categories protein binding and unfolded protein binding. The KEGG category pathways in cancer also overlap with these GO categories.
- The three KEGG pathways oxidative phosphorylation, Huntington's disease and Parkinson's disease, together with three GO Molecular Function categories (hydrogen ion transmembrane transporter activity, inorganic cation transmembrane transporter activity, and monovalent inorganic cation transmembrane transporter activity), which include four to six common genes.
- The two KEGG pathways extracellular matrix receptor interaction and focal adhesion, with all six genes in these two KEGG categories also included in the large GO Molecular Function category protein binding.

**Table 3:** Consistently enriched GO and KEGG categories.

ID	Category	Number of genes in category	54 gene list		124 gene list		1475 gene list	
GO Biological Process (8 tools)			Tools	Genes	Tools	Genes	Tools	Genes
**GO:0008283**	**cell proliferation**	1166	0	-	4	22	8	175
**GO:0048518**	**positive regulation of biological process**	2252	3	18	3	31	5	258
**GO:0048522**	**positive regulation of cellular process**	2050	3	18	3	30	5	243
**GO:0042981**	**regulation of apoptosis**	892	2	10	3	18	5	129
**GO:0042127**	**regulation of cell proliferation**	853	0	-	3	17	6	121
**GO:0042221**	**response to chemical stimulus**	1520	1	13	4	31	6	250
**GO Molecular Function (7 tools)**								
**GO:0015078**	**hydrogen ion transmembrane transporter activity**	93	0	-	3	6	6	29
**GO:0022890**	**inorganic cation transmembrane transporter activity**	216	0	-	3	6	5	36
**GO:0015077**	**monovalent inorganic cation transmembrane transporter activity**	172	0	-	3	6	6	30
**GO:0005515**	**protein binding**	8184	3	38	5	80	6	843
**GO:0051082**	**unfolded protein binding**	113	4	4	1	4	7	30
**KEGG pathway (7 tools)**								
**KEGG4512**	**ECM-receptor interaction**	58	2	2	3	5	4	24
**KEGG4510**	**focal adhesion**	135	2	3	2	5	5	49
**KEGG5016**	**Huntington's disease**	143	0	-	2	6	4	41
**KEGG190**	**oxidative phosphorylation**	203	1	2	5	7	4	33
**KEGG5200**	**pathways in cancer**	241	2	4	2	8	4	67
**KEGG5012**	**Parkinson's disease**	109	0	-	2	5	4	34
**KEGG5222**	**small cell lung cancer**	65	0	-	2	3	4	23

In each case, the number of enrichment tools reporting the category as significantly overrepresented and the maximal number of genes from the category present in the input gene list are indicated.

**Table 4:** Overlap of the genes from the consistently enriched GO and KEGG categories in the GEP studies on prognosis of CRC.

	ID	Category		a§	b§	c§	d§	e§	f§	g*	h*	i*	j	k§	l#	m#	n*	o*	p§	q*	r
			Number genes	22	31	30	18	17	31	6	6	6	80	4	5	5	6	7	8	5	3
**a** §	**GO:0008283**	**cell proliferation**	**22**		13	13	9	17	11	-	-	-	20	2	1		1	-	5	-	2
**b** §	**GO:0048518**	**positive regulation of biological process**	**31**			29	17	14	16	-	-	-	29	3	2	1	1	-	6	-	2
**c** §	**GO:0048522**	**positive regulation of cellular process**	**30**				15	12	16	-	-	-	27	3	2	1	1	-	5	-	2
**d** §	**GO:0042981**	**regulation of apoptosis**	**18**					7	9	-	-	-	18	2	1	1	1	-	4	-	2
**e** §	**GO:0042127**	**regulation of cell proliferation**	**17**						9	-	-	-	16	1	1	-	1	-	5	-	2
**f** §	**GO:0042221**	**response to chemical stimulus**	**31**							1	1	1	26	2	2	2	2	1	5	1	1
**g** *	**GO:0015078**	**hydrogen ion transm. transporter activity**	**6**								6	6	1	-	-	-	4	6	-	4	-
**h** *	**GO:0022890**	**inorganic cation transm. transp. activity**	**6**									6	3	-	-	-	4	6	-	4	-
**i** *	**GO:0015077**	**monovalent inorg. cation transm. transp. activity**	**6**										3	-	-	-	4	6	-	4	-
**j**	**GO:0005515**	**protein binding**	**80**											4	5	5	1	1	6		2
**k** §	**GO:0051082**	**unfolded protein binding**	**4**												-	-	-	-	-	-	-
**l** #	**KEGG4512**	**ECM-receptor interaction**	**5**													4	-	-	1	-	1
**m** #	**KEGG4510**	**focal adhesion**	**5**														-	-	1	-	1
**n** *	**KEGG5016**	**Huntington's disease**	**6**															5	-	5	-
**o** *	**KEGG190**	**oxidative phosphorylation**	**7**																-	5	-
**p** §	**KEGG5200**	**pathways in cancer**	**8**																	-	2
**q** *	**KEGG5012**	**Parkinson's disease**	**5**																		-
**r**	**KEGG5222**	**small cell lung cancer**	**3**																		

The number of genes from the 124 gene list belonging to each category is indicated, as well as the number of overlapping genes between each pair of categories. The three biologically meaningful category groups identified are marked:

§ , cell proliferation and apoptosis;

*, oxidative phosphorylation;

# , extracellular matrix receptor interaction.

Enrichment analysis using the Ingenuity software confirmed the results obtained with the GO and KEGG databases. The only overrepresented canonical pathway in the 124 gene list was oxidative phosphorylation (P_corrected_ = 2.7×10^−2^), while this category was the third most significant pathway (P_corrected_ = 1.0×10^−5^) among the 159 enriched canonical pathways in the 1475 gene set. The results for the Bio Function categories were too unspecific, due to the large number of enriched categories reported for each of the three gene lists (61 to 77 enriched terms) (Table 2). However, the general categories cell death, cancer and cellular growth and proliferation were among the top four enriched terms in the three gene lists, with corrected P values between 10^−4^ and 10^−20^ (data not shown).

Enrichment analysis with all enrichment tools was also performed individually for the four single GEP studies reporting more than 100 unique mapped genes [13]–[16] (Table S7). From the 18 GO/KEGG terms, the general GO categories were reported only by some of the four individual GEP studies, while the more specific KEGG pathways appeared to be more commonly reported. In the GEP study from Bertucci et al. [14] almost all 18 categories came out as overrepresented in the gene list.

## Discussion

The large number of published microarray studies on prognosis of CRC, showing a very low overlap in results, has provided no generally accepted gene expression profile for prediction of CRC prognosis. Additionally, no genome-wide association studies of outcome in CRC have been published, but are now underway [3]. The heterogeneity in the GEP study design regarding the features related to disease progression makes a consistent comparison of results between the single studies very difficult [17]. Here, we report the results of our approach, in which we used the largest collection of GEP studies on CRC prognosis so far, and for the first time applied and compared several enrichment tools to the extracted gene lists. This strategy allowed us to identify the oxidative phosphorylation chain and the extracellular matrix receptor interaction categories, as well as a general category related to cell proliferation and apoptosis, as the only significantly and consistently overrepresented pathways involved in CRC progression.

In the first part of the study, we tried to overcome the lack of reproducibility in the GEP studies on CRC prognosis by selecting the genes reported in more than one study, in an attempt to reduce false positive results. From a total of 1475 unique, annotated genes identified in 23 independent GEP studies, 124 genes (8.4%) were reported in at least two studies, and only 9 of them (0.6%) in three studies, which give us a clear idea of the lack of reproducibility at the individual gene level. This lack of reproducibility does not seem to be caused by the different investigated features related to cancer prognosis, since the proportion of genes reported by two studies of the same class (2.8% for recurrence, 5.3% for metastasis studies, and 0% for survival studies) was even lower than for all studies together (8.4%). Unexpectedly, 70 out of these 124 genes (56.5%) showed contrasting direction in expression change between two single studies, while for the other 54 (43.5%) the expression change was in the same direction, 19 up-regulated (15.3%) and 35 down-regulated (28.2%). The proportion of up- and down –regulated genes was approximately the same also within each of the consistently enriched GO and KEGG categories. The inconsistencies in the direction of differential expression can be attributed to several factors: first, the large number of false positives observed in microarray gene expression studies [18]; second, overgeneralization of comparisons in meta-analyses, especially related to experimental design and background reference for expression; third, heterogeneity in the tissue samples used in each study; and fourth, inaccurate results due to poor study design [19]. However, a clear explanation for these discrepancies is missing. Only one previous meta-analysis of ten GEP studies has reported a list of 13 genes differentially expressed in CRC with good versus bad prognosis, reported by at least two independent studies [4]. A comparison with our results showed that eight of the genes are also present in our 124 gene list, with the same direction in expression change (IGF2, IQGAP1, YWHAH, DEK, TP53, OAS1, RARB, and PDCD10), three of them (IGF2, TP53 and RARB) belonging to the group of broad categories related to cell proliferation and apoptosis. The other five genes reported by Cardoso et al. were actually not present in one of the two GEP studies mentioned in the meta-analysis.

The second part of our analysis made use of freely available enrichment tools to detect which GO categories or KEGG pathways were significantly overrepresented in the three gene sets obtained from the 23 gene expression profiling studies (1475, 124 or 54 gene list). Here, we attempted to overcome the known differences in the overrepresentation analysis results by using up to ten different singular enrichment analysis (SEA or class I) enrichment tools. We selected only those categories which were reported to be overrepresented by several tools and in at least two of the three gene lists as consistently enriched categories. Gene set enrichment analysis tools (GSEA or class II) were not considered, since they require a summarized biological value (e.g. expression fold change) for each of the genes in the input, which was not available for most of the studies. Recently developed modular enrichment analysis tools (MEA or class III) consider inter-relationships of GO terms, but they require relatively large gene input lists for a biologically meaningful analysis [6], and this was not the case in our study.

Enrichment tools suffer from several limitations, which have been described in detail elsewhere [6], [7], and it is recommended to test multiple tools, even if they have similar capabilities and functions [7]. For example, it has been observed that for the same input data, ten different ontological analysis programs resulted in P values ranging several orders of magnitude for some GO terms [7]; the same effect was observed in our study. KEGG pathways represent relatively well-defined known biological pathways, rather than the more broad GO categories. The use of pathway classification tools is anyway not free of difficulties [20]. A recent overrepresentation analysis of pathways from genome-wide association study data also reported differences in outcome between three of the pathway enrichment tools we used (DAVID, GATHER and WebGestalt) [20]. Factors that can cause these differences in results include: the sources and versions of annotation files; the statistical model applied for the enrichment analysis; the set of reference genes against which the P values for each term in the results are calculated; and the method of correction for multiple experiments [21]. In our analysis, the whole genome was used as a background reference, and a significance threshold of corrected P value<0.05 was used in all analyses. Despite this uniformity in the conditions used, we also observed a considerable variability in the number of reported enriched categories and in the P values. Thus, this variability can be attributed to the statistical model applied for the enrichment analysis, to the method of correction for multiple testing, and to differences in the versions of the GO and KEGG data sources used. However, and despite this apparent variation, most of the enriched categories reported by the more stringent tools were included in the ones reported by those tools reporting a larger number of terms, demonstrating the utility of our study strategy. Thus, bioinformatic enrichment tools are a powerful approach to identify biological processes in high-throughput data analysis, but selection of enriched categories based on only one enrichment tool appears to be quite arbitrary.

Finally, after application of rigorous selection criteria, a total of 18 categories (11 GO terms and seven KEGG pathways) were considered as consistently overrepresented in the gene lists extracted from the 23 different GEP studies on CRC prognosis. In the 124 gene list, a very high overlap of genes among the 18 categories was observed, reducing the number of categories with biological significance to three clearly different groups. First, a very general group related to cell proliferation, apoptosis and protein binding, which included a high proportion of the genes from each of the three gene sets. Second, and more interesting, the oxidative phosphorylation chain, including seven genes (ATP5C1, ATP6AP1, ATP6V1H, COX5B, COX6B1, NDUFA1, and UQCRC1) (Figure S1), five of them shared with Huntington's and Parkinson's disease KEGG categories. Already several decades ago, it was suggested that impaired oxidative metabolism may cause malignant growth [22]. This assumption, known as Warburg's hypothesis, has been rediscovered by a broad range of experimental approaches showing interaction of mitochondrial metabolism and tumour growth [23], [24]. Added to that, germline mutations in the mitochondrial succinate dehydrogenase (complex II of the oxidative phosphorylation chain) subunits SDHD, SDHC, and SDHB are a frequent cause of paragangliomas of the head and neck and of phaeochromocytomas [25]. Also Huntington's and Parkinson's disease, the other two enriched KEGG pathways with genes from the oxidative phosphorylation chain, are associated with mitochondrial dysfunction [26]. Third, both KEGG terms extracellular matrix receptor interaction and focal adhesion included four common genes (COL5A1, FN1, SPP1, and TNXB) (Figure S2). Specific interactions of the extracellular matrix molecules control cellular activities such as adhesion, differentiation, apoptosis and proliferation [27]. Thus, and based on the functional classes of the genes, they look promising for studies aimed to investigate their possible influence on the prognosis of CRC. Especially, the KEGG pathways oxidative phosphorylation, extracellular matrix receptor interaction and focal adhesion may provide new targets for drug development. Six of the 23 independent GEP studies performed an enrichment analysis of GO and/or KEGG categories with their list of differentially expressed genes, in all cases using only one enrichment tool. Only the GEP study from Jorissen et al. [16] reported two KEGG pathways also reported in our analysis (ECM-receptor interaction and focal adhesion). When we searched for overrepresented categories in individual GEP studies, clear differences between the studies were observed. Although terms of specific KEGG pathways oxidative phosphorylation and extracellular matrix molecules were commonly reported, the general GO terms reported in our global approach were identified only by some of the studies. These results show that our approach of combining the data of 23 individual GEP studies not only is able to identify the common pathways reported by individual large studies, but it is also able to report novel consistently overrepresented pathways, which may be lost in small studies.

In conclusion, our pathway-based enrichment analysis of 23 independent gene expression profiling studies on prognosis of CRC indicated the oxidative phosphorylation chain, the extracellular matrix receptor interaction category, and a general category related to cell proliferation and apoptosis as significantly and consistently overrepresented prognostic categories for CRC. These categories have been functionally clearly related with cancer progression, and deserve further investigation. It would be of special interest if future GEP studies performed in large sample cohorts could validate our results and identify these categories as classifiers for bad prognosis.

## Materials and Methods

### Gene expression profiling (GEP) studies

A total of 27 GEP studies for prognosis prediction of CRC were included in the analysis (Table 1): the 16 GEP studies named in two recent reviews [2], [3], three additional studies included in a meta-analysis [4], and eight more recent studies (PubMed search from January 2009 till March 2010) not included in the previous reviews/meta-analysis. Four of the 27 studies used partially overlapping samples [28]–[31], and another study [32] was actually a follow-up of a previous one [33], reducing the total number of independent studies to 23. According to the investigated feature related to disease progression, seven of the studies were based on the existence of recurrence, thirteen on the presence of metastasis, two on survival data, and one on a combination of survival and recurrence data. Due to the heterogeneous nature of the available data, no attempt was made to perform quantitative meta-analyses.

### Gene set collection

It has been reported that the type of gene identifier used to specify the differentially regulated genes can potentially affect the results of the subsequent analysis [21]. We used the official HUGO gene symbol as a consistent identifier for the reported genes. If the gene symbol was not reported in the GEP study, we used the following tools to convert the reported identifiers into the gene symbol: NetAffx from Affymetrix (www.affymetrix.com), EntrezGene from NCBI (www.ncbi.nlm.nih.gov/gene/), and the Gene ID conversion tool from the DAVID bioinformatics resources [34]. In many cases, the number of gene identifiers (IDs) reported by the GEP study did not actually correspond to the annotated genes, but to probes on the expression array or GenBankIDs. Added to that, several studies counted some genes more than once. Therefore, the current number of annotated genes finally used was lower than the one reported by the majority of the GEP studies (Table 1).

### Gene lists

The lists of annotated genes reported by each of the 23 independent GEP studies for prognosis of CRC included in the analysis were combined in order to identify those genes reported in two or more studies. Three different gene lists were considered for the subsequent enrichment analysis: all unique, annotated genes reported (1475 genes) (Table S2); those genes reported in at least two GEP studies (124 genes) (Table S1); and the ones which additionally showed the same direction in gene expression change, either up- or down-regulation, in two GEP studies (54 genes) (Table S1).

### Enrichment analysis

We performed enrichment analyses using the databases GO (Biological Process and Molecular Function) and KEGG pathways. For all enrichment tools, the input gene set consisted of the 1475 gene list, the 124 gene list, or the 54 gene list, respectively.

Ten enrichment software tools (see URLs) were selected based on their freeware availability, their frequent appearance in recent publications and their user-friendly application. Default options were applied in all tools, with a significance threshold of 0.05 for adjusted P value, at least two genes from the input list in the enriched category, and the whole genome as a reference background. For GATHER, the recommended ln(Bayes factor) >6 was used as significance threshold. The Ingenuity software makes use of its own two databases, Top Bio Function and Top Canonical Pathways, which however are comparable with the GO and the KEGG databases, respectively, used by the other enrichment tools. Key statistical and multiple testing correction methods used by each tool are shown in Table S8.

### Consistently enriched categories

Only the GO or KEGG categories reported to be significantly enriched by several enrichment tools in a gene list were considered as consistently overrepresented. In an attempt to select only top-ranked categories, we took into account the size differences between GO and KEGG categories as well as the differences in the number of categories reported by each tool. The number of tools established as a threshold was, for each gene list and GO or KEGG databases, the one reporting at least five common enriched categories for that number of tools (Table S6). For both the 54 and the 124 gene list, the threshold was three enrichment tools for GO Biological Process and Molecular Function, and two enrichment tools for KEGG pathways. For the 1475 gene list, the threshold was five enrichment tools for GO Biological Process and Molecular Function, and four enrichment tools for KEGG pathways (Table S6). Since the three gene lists are related (the 54 gene list is included in the 124 gene list, which is part of the 1475 gene list), we additionally selected the categories reported to be enriched in the large 1475 gene list and at least one of the other two lists. With this double filter, we guaranteed that the survived enriched categories are the ones consistently enriched in the GEP studies on prognosis of CRC.

## Supporting Information

Figure S1 Representation of the KEGG oxidative phosphorylation pathway (map00190), with the seven genes from the 124 gene list indicated in red, as well as the location of the four complexes in the mitochondrial electron-transport chain to which they belong.
(TIF)

Figure S2 Representation of the KEGG ECM-receptor interaction category (map04512), with location of the fives genes from the 124 gene list indicated in red.
(TIF)

Table S1 124 genes reported in at least two gene expression profiling studies on CRC prognosis.
(DOC)

Table S2 1475 unique, annotated genes reported in 23 independent gene expression profiling studies on CRC prognosis.
(DOC)

Table S3 Results of all enrichment tools used with the 54 gene list. Only those categories selected by at least two enrichment tools are shown. In each case, the first row represents the overrepresentation P value adjusted for multiple testing, and the second row the number of genes in the category within the 54 gene list. Table S3A. Results for Gene Ontology Biological Process categories; Table S3B. Results for Gene Ontology Molecular Function categories; Table S3C. Results for KEGG pathway categories.
(DOC)

Table S4 Results of all enrichment tools used with the 124 gene list. Only those categories selected by at least two enrichment tools are shown. In each case, the first row represents the overrepresentation P value adjusted for multiple testing, and the second row the number of genes in the category within the 124 gene list. Table S4A. Results for Gene Ontology Biological Process categories; Table S4B. Results for Gene Ontology Molecular Function categories; Table S4C. Results for KEGG pathway categories.
(DOC)

Table S5 Results of all enrichment tools used with the 1475 gene list. Only those categories selected by at least two enrichment tools are shown. In each case, the first row represents the overrepresentation P value adjusted for multiple testing, and the second row the number of genes in the category within the 1475 gene list. Table S5A. Results for Gene Ontology Biological Process categories; Table S5B. Results for Gene Ontology Molecular Function categories; Table S5C. Results for KEGG pathway categories.
(DOC)

Table S6 Number of overrepresented GO and KEGG categories reported by more than one enrichment tool.
(DOC)

Table S7 Result of the enrichment analysis in four individual GEP studies for the consistently enriched GO and KEGG categories of the global analysis.
(DOC)

Table S8 Enrichment tools used and their characteristics.
(DOC)
