# Supplementary figures and images for: Consensus Pathways Implicated in Prognosis of Colorectal Cancer Identified Through Systematic Enrichment Analysis of Gene Expression Profiling Studies
Source: PLoS One. 2011 Apr 25;6(4):e18867. doi: 10.1371/journal.pone.0018867 (PMC3081819; doi:10.1371/journal.pone.0018867)

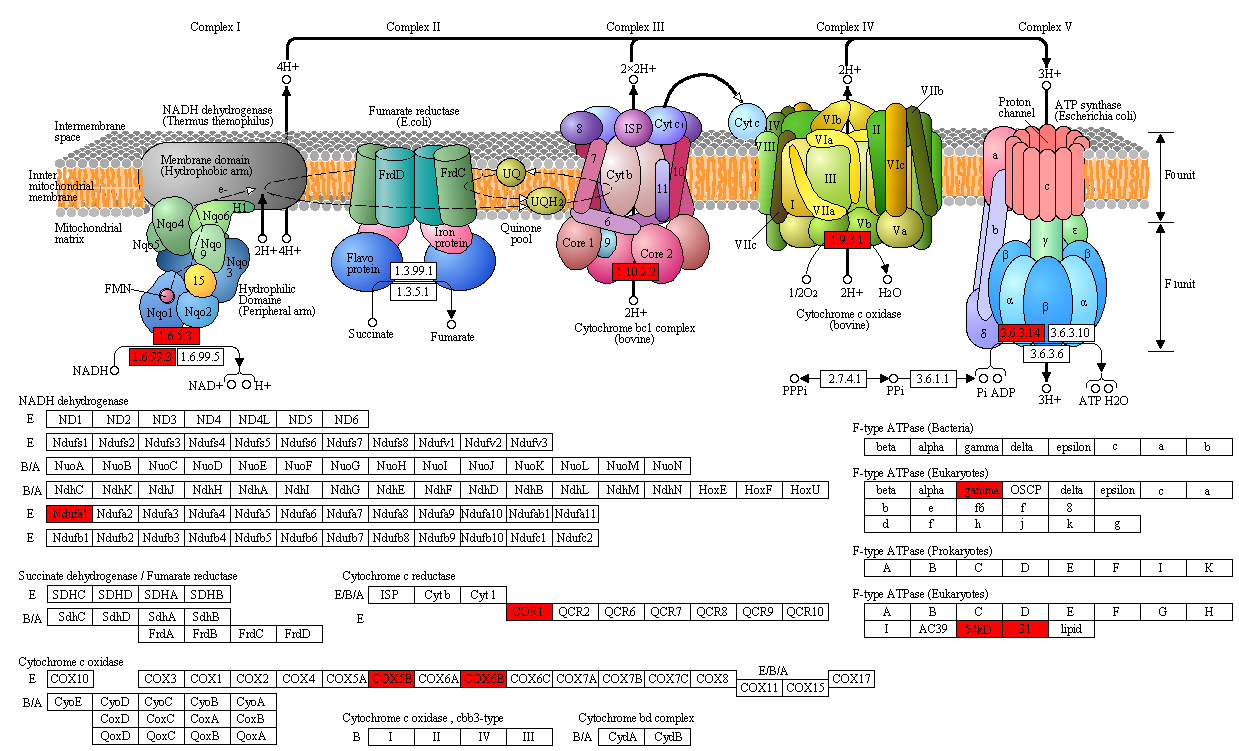

Supplement: Figure S1 — Representation of the KEGG oxidative phosphorylation pathway (map00190), with the seven genes from the 124 gene list indicated in red, as well as the location of the four complexes in the mitochondrial electron-transport chain to which they belong. (TIF) [file pone.0018867.s001.tif]

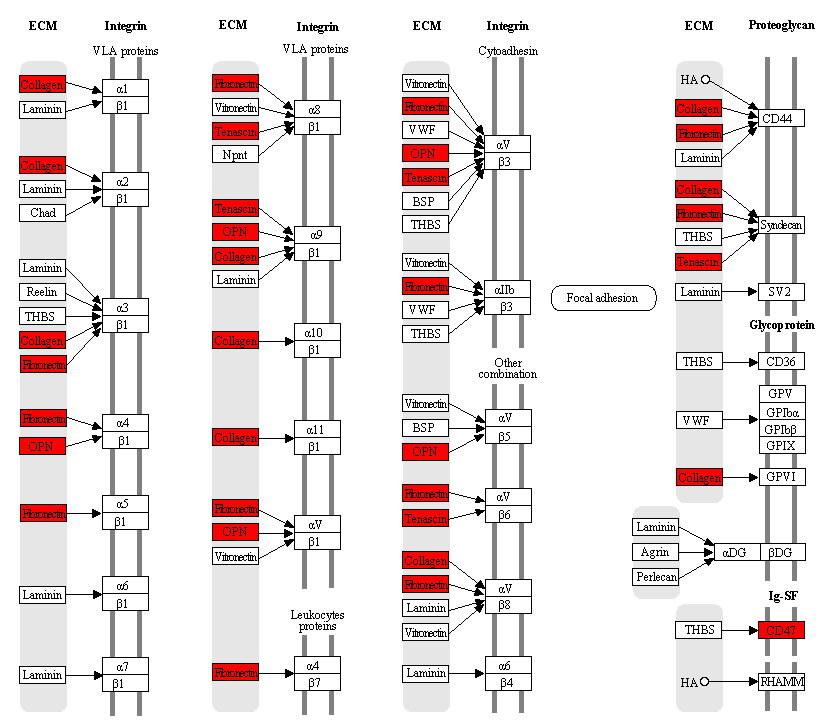

Supplement: Figure S2 — Representation of the KEGG ECM-receptor interaction category (map04512), with location of the fives genes from the 124 gene list indicated in red. (TIF) [file pone.0018867.s002.tif]
