# Supplementary material for: Consensus Pathways Implicated in Prognosis of Colorectal Cancer Identified Through Systematic Enrichment Analysis of Gene Expression Profiling Studies
Source: PLoS One. 2011 Apr 25;6(4):e18867. doi: 10.1371/journal.pone.0018867 (PMC3081819; doi:10.1371/journal.pone.0018867)
Supplement: Table S1 — 124 genes reported in at least two gene expression profiling studies on CRC prognosis. (DOC) [file pone.0018867.s003.doc]

**Table S1. 124 genes reported in at least two gene expression profiling studies on CRC prognosis.**

| **Gene symbol** | **Common genes in at least two gene expression profiling studies of the same class** | **Common genes in a biologically meaningful enriched group of categories** | **Up/Down regulated in poor prognosis samples in at least two gene expression profiling studies** |
| --- | --- | --- | --- |
| ACTB |  | c) | up |
| ADAMTS12 |  |  | up |
| APOC1 |  |  | up |
| ATP5C1* | metastasis | b) | up |
| CES2 |  |  | up |
| DACT1 |  |  | up |
| FAS | recurrence | a) | up |
| FN1* | metastasis | c) | up |
| HEYL |  |  | up |
| HSP90AB1* | metastasis | a) | up |
| IQGAP1* | recurrence |  | up |
| MTA2 | metastasis |  | up |
| PLA2G16 | metastasis |  | up |
| SERPINA1 | metastasis |  | up |
| SPARC | metastasis |  | up |
| SPP1*# | metastasis | a), c) | up |
| TIMP1 | metastasis | a) | up |
| VCAN | metastasis |  | up |
| VDR |  | a) | up |
| ARPP19 |  |  | down |
| ATP6V1H | metastasis | b) | down |
| C7orf44 | recurrence |  | down |
| CA2* | metastasis |  | down |
| CALR | recurrence | a) | down |
| CASP1 | metastasis | a) | down |
| CDC37 |  |  | down |
| CYP51A1*# | recurrence |  | down |
| DEK |  |  | down |
| EIF2AK2 | metastasis |  | down |
| EPRS |  |  | down |
| FRYL | metastasis |  | down |
| HSPD1 |  | a) | down |
| IGHA1 | metastasis |  | down |
| KCNAB1 |  |  | down |
| KLF4 |  | a) | down |
| MBD4 | recurrence |  | down |
| MGC29506 | metastasis |  | down |
| NFE2L1 | recurrence |  | down |
| OAZ1 |  |  | down |
| OLA1 | metastasis |  | down |
| PERP |  |  | down |
| PPP1R15A |  |  | down |
| PSMA6 |  |  | down |
| RPS5* | metastasis |  | down |
| SELENBP1 | metastasis |  | down |
| SLC39A8 | metastasis |  | down |
| TAGLN2 |  |  | down |
| TXN* | metastasis |  | down |
| UBQLN4 |  |  | down |
| WNT2 | metastasis | a) | down |
| WSCD1 |  |  | down |
| XRCC5 | metastasis | a) | down |
| YWHAH | recurrence |  | down |
| ZC3H12A | metastasis |  | down |
| AMD1 |  |  | both |
| AMPD1 |  |  | both |
| ATP6AP1 |  | b) | both |
| B2M | metastasis | a) | both |
| BSG |  |  | both |
| CCL20 | metastasis |  | both |
| CD47 |  | a), c) | both |
| CDK10 |  |  | both |
| CHD2 |  |  | both |
| CKS2 | metastasis |  | both |
| COL5A1 | metastasis | c) | both |
| COX5B |  | b) | both |
| COX6B1 | metastasis | b) | both |
| CXCL6 | recurrence |  | both |
| DFNB31 |  |  | both |
| ERP29 |  |  | both |
| FOXO1 | recurrence | a) | both |
| GCG |  |  | both |
| GDI2 |  |  | both |
| H2AFZ |  |  | both |
| HNRNPC | metastasis |  | both |
| HNRNPM |  |  | both |
| IGF2 | metastasis | a) | both |
| IL8 |  | a) | both |
| ITM2A |  |  | both |
| LRMP |  |  | both |
| LTBP3 | metastasis |  | both |
| MAOA |  |  | both |
| MGP |  |  | both |
| MRPL37 |  |  | both |
| NDUFA1 | metastasis | b) | both |
| NME1 | metastasis | a) | both |
| NTM |  |  | both |
| OAS1 |  |  | both |
| PBK |  |  | both |
| PDCD10 | recurrence |  | both |
| PHB | metastasis |  | both |
| POSTN |  |  | both |
| PRSS8 |  |  | both |
| PSMC2 |  |  | both |
| PTGDS | metastasis |  | both |
| RAB27A |  |  | both |
| RARB |  | a) | both |
| RBMS1 | recurrence |  | both |
| REG1B | metastasis |  | both |
| RPL31 | metastasis |  | both |
| RPS12 |  |  | both |
| RPS15A | metastasis | a) | both |
| S100P |  |  | both |
| SFRS1 |  |  | both |
| SLC4A4 |  |  | both |
| SMAD6 | metastasis |  | both |
| SRP72 |  |  | both |
| SSBP1 | metastasis |  | both |
| TARS |  |  | both |
| TCF3 | metastasis |  | both |
| TIMM13 |  |  | both |
| TM4SF1 | metastasis |  | both |
| TMCC1 | metastasis |  | both |
| TNXB |  | c) | both |
| TP53 |  | a) | both |
| TRIM26 | metastasis |  | both |
| TUBB2A | metastasis |  | both |
| UBD | metastasis |  | both |
| UQCRC1 | metastasis | b) | both |
| VPS16 | recurrence |  | both |
| WWTR1 | metastasis |  | both |
| ZFAND1 | recurrence |  | both |
| ZNF117 | metastasis |  | both |
| ZNF236 | recurrence |  | both |

*Nine genes reported in three GEP studies; #Two genes with same direction in expression change in the three GEP studies; a) Large group of categories related to cell proliferation, apoptosis and protein binding; only genes present in at least five of the nine categories (six GO Biological Process, two GO Molecular Function, and one KEGG category); b) Oxidative phosphorylation and related categories (three GO Molecular function and three KEGG categories); c) Extracellular matrix receptor interaction and related categories (one GO Molecular Function and two KEGG categories).
