# Supplementary material for: Consensus Pathways Implicated in Prognosis of Colorectal Cancer Identified Through Systematic Enrichment Analysis of Gene Expression Profiling Studies
Source: PLoS One. 2011 Apr 25;6(4):e18867. doi: 10.1371/journal.pone.0018867 (PMC3081819; doi:10.1371/journal.pone.0018867)
Supplement: Table S2 — 1475 unique, annotated genes reported in 23 independent gene expression profiling studies on CRC prognosis. (DOC) [file pone.0018867.s004.doc]

**Table S2. 1475 unique, annotated genes reported in 23 independent gene expression profiling studies on CRC prognosis.**

| **Gene symbol** | **Number of gene expression profiling studies reporting the gene** | **Up/Down regulated in poor prognosis samples** |
| --- | --- | --- |
| SPP1 | 3 | up |
| CYP51A1 | 3 | down |
| ATP5C1 | 3 | both |
| CA2 | 3 | both |
| FN1 | 3 | both |
| HSP90AB1 | 3 | both |
| IQGAP1 | 3 | both |
| RPS5 | 3 | both |
| TXN | 3 | both |
| ACTB | 2 | up |
| ADAMTS12 | 2 | up |
| APOC1 | 2 | up |
| CES2 | 2 | up |
| COL10A1 | 2 | up |
| DACT1 | 2 | up |
| FAS | 2 | up |
| HEYL | 2 | up |
| MTA2 | 2 | up |
| PLA2G16 | 2 | up |
| SERPINA1 | 2 | up |
| SPARC | 2 | up |
| TIMP1 | 2 | up |
| VCAN | 2 | up |
| VDR | 2 | up |
| ARPP19 | 2 | down |
| ATP6V1H | 2 | down |
| C7orf44 | 2 | down |
| CASP1 | 2 | down |
| CDC37 | 2 | down |
| CHMP2A | 2 | down |
| DEK | 2 | down |
| EIF2AK2 | 2 | down |
| EPRS | 2 | down |
| FRYL | 2 | down |
| HSPD1 | 2 | down |
| IGHA1 | 2 | down |
| KCNAB1 | 2 | down |
| KLF4 | 2 | down |
| MBD4 | 2 | down |
| MGC29506 | 2 | down |
| NFE2L1 | 2 | down |
| OAZ1 | 2 | down |
| OLA1 | 2 | down |
| PERP | 2 | down |
| PPP1R15A | 2 | down |
| PSMA6 | 2 | down |
| SELENBP1 | 2 | down |
| SLC39A8 | 2 | down |
| TAGLN2 | 2 | down |
| UBQLN4 | 2 | down |
| WNT2 | 2 | down |
| WSCD1 | 2 | down |
| XRCC5 | 2 | down |
| YWHAH | 2 | down |
| ZC3H12A | 2 | down |
| AMD1 | 2 | both |
| AMPD1 | 2 | both |
| ATP6AP1 | 2 | both |
| B2M | 2 | both |
| BSG | 2 | both |
| CCL20 | 2 | both |
| CD47 | 2 | both |
| CDK10 | 2 | both |
| CHD2 | 2 | both |
| CKS2 | 2 | both |
| COL5A1 | 2 | both |
| COX5B | 2 | both |
| COX6B1 | 2 | both |
| CXCL6 | 2 | both |
| DFNB31 | 2 | both |
| ERP29 | 2 | both |
| FOXO1 | 2 | both |
| GCG | 2 | both |
| GDI2 | 2 | both |
| H2AFZ | 2 | both |
| HNRNPC | 2 | both |
| HNRNPM | 2 | both |
| IGF2 | 2 | both |
| IL8 | 2 | both |
| ITM2A | 2 | both |
| LRMP | 2 | both |
| LTBP3 | 2 | both |
| MAOA | 2 | both |
| MGP | 2 | both |
| MRPL37 | 2 | both |
| NDUFA1 | 2 | both |
| NME1 | 2 | both |
| NTM | 2 | both |
| OAS1 | 2 | both |
| PBK | 2 | both |
| PDCD10 | 2 | both |
| PHB | 2 | both |
| POSTN | 2 | both |
| PRSS8 | 2 | both |
| PSMC2 | 2 | both |
| PTGDS | 2 | both |
| RAB27A | 2 | both |
| RARB | 2 | both |
| RBMS1 | 2 | both |
| REG1B | 2 | both |
| RPL31 | 2 | both |
| RPS12 | 2 | both |
| RPS15A | 2 | both |
| S100P | 2 | both |
| SFRS1 | 2 | both |
| SLC4A4 | 2 | both |
| SMAD6 | 2 | both |
| SRP72 | 2 | both |
| SSBP1 | 2 | both |
| TARS | 2 | both |
| TCF3 | 2 | both |
| TIMM13 | 2 | both |
| TM4SF1 | 2 | both |
| TMCC1 | 2 | both |
| TNXB | 2 | both |
| TP53 | 2 | both |
| TRIM26 | 2 | both |
| TUBB2A | 2 | both |
| UBD | 2 | both |
| UQCRC1 | 2 | both |
| VPS16 | 2 | both |
| WWTR1 | 2 | both |
| ZFAND1 | 2 | both |
| ZNF117 | 2 | both |
| ZNF236 | 2 | both |
| ABCA5 | 1 | up |
| ABCC2 | 1 | up |
| ABCC9 | 1 | up |
| ABCD1 | 1 | up |
| ABP1 | 1 | up |
| ACYP2 | 1 | up |
| ADAM28 | 1 | up |
| ADO | 1 | up |
| AGER | 1 | up |
| AGXT | 1 | up |
| AHI1 | 1 | up |
| AHNAK2 | 1 | up |
| AK1 | 1 | up |
| AKAP12 | 1 | up |
| AKAP13 | 1 | up |
| ALDH3A2 | 1 | up |
| ALDH9A1 | 1 | up |
| AMN | 1 | up |
| AMOTL1 | 1 | up |
| ANG | 1 | up |
| ANKRD18A | 1 | up |
| ANXA11 | 1 | up |
| ANXA2 | 1 | up |
| ANXA3 | 1 | up |
| AP3D1 | 1 | up |
| APLP2 | 1 | up |
| ARAP1 | 1 | up |
| ARHGAP4 | 1 | up |
| ARHGAP5 | 1 | up |
| ARL1 | 1 | up |
| ARL17A | 1 | up |
| ASMTL | 1 | up |
| ASPN | 1 | up |
| ATF2 | 1 | up |
| ATF7IP2 | 1 | up |
| ATG12 | 1 | up |
| ATG5 | 1 | up |
| ATP1A1 | 1 | up |
| ATP2A2 | 1 | up |
| ATP2B4 | 1 | up |
| ATP5B | 1 | up |
| ATP6V01 | 1 | up |
| ATP6V0E1 | 1 | up |
| ATP6V1G1 | 1 | up |
| ATXN7 | 1 | up |
| B4GALT3 | 1 | up |
| BAMBI | 1 | up |
| BBS9 | 1 | up |
| BCL11A | 1 | up |
| BGN | 1 | up |
| BHLHB2 | 1 | up |
| BHLHB3 | 1 | up |
| BICC1 | 1 | up |
| BIRC2 | 1 | up |
| BMP2 | 1 | up |
| BOP1 | 1 | up |
| BRAP | 1 | up |
| BRE | 1 | up |
| BTF3 | 1 | up |
| C10orf10 | 1 | up |
| C11orf47 | 1 | up |
| C12orf4 | 1 | up |
| C14orf108 | 1 | up |
| C14orf2 | 1 | up |
| C1orf21 | 1 | up |
| C1orf9 | 1 | up |
| C4orf31 | 1 | up |
| C5AR1 | 1 | up |
| C5orf23 | 1 | up |
| C5orf41 | 1 | up |
| C6orf108 | 1 | up |
| C6orf64 | 1 | up |
| CALCOCO2 | 1 | up |
| CAMKK2 | 1 | up |
| CASP3 | 1 | up |
| CCDC121 | 1 | up |
| CCND1 | 1 | up |
| CCND2 | 1 | up |
| CCNF | 1 | up |
| CCT3 | 1 | up |
| CD1A | 1 | up |
| CD44 | 1 | up |
| CD48 | 1 | up |
| CD55 | 1 | up |
| CD68 | 1 | up |
| CD74 | 1 | up |
| CD99 | 1 | up |
| CDC25B | 1 | up |
| CDC42 | 1 | up |
| CDH2 | 1 | up |
| CDH5 | 1 | up |
| CEACAM7 | 1 | up |
| CENPB | 1 | up |
| CEP70 | 1 | up |
| CFB | 1 | up |
| CFH | 1 | up |
| CFL1 | 1 | up |
| CHGA | 1 | up |
| CIAO1 | 1 | up |
| CIAPIN1 | 1 | up |
| CKS1B | 1 | up |
| CLDN11 | 1 | up |
| CLEC11A | 1 | up |
| CLIC2 | 1 | up |
| CLIC4 | 1 | up |
| CLINT1 | 1 | up |
| CLTA | 1 | up |
| CNDP2 | 1 | up |
| CNNM4 | 1 | up |
| CNPY2 | 1 | up |
| COL1A2 | 1 | up |
| COL27A1 | 1 | up |
| COL8A1 | 1 | up |
| COMP | 1 | up |
| COPG | 1 | up |
| COPS4 | 1 | up |
| COPS5 | 1 | up |
| COPS7B | 1 | up |
| COPS8 | 1 | up |
| COX10 | 1 | up |
| COX4I1 | 1 | up |
| COX5A | 1 | up |
| COX6C | 1 | up |
| CPA3 | 1 | up |
| CPB1 | 1 | up |
| CREBL2 | 1 | up |
| CSNK1E | 1 | up |
| CTSK | 1 | up |
| CXCL1 | 1 | up |
| CXCL10 | 1 | up |
| CXCL11 | 1 | up |
| CXCL12 | 1 | up |
| CXCL2 | 1 | up |
| CXCL9 | 1 | up |
| CXCR7 | 1 | up |
| CYP17A1 | 1 | up |
| CYP1B1 | 1 | up |
| CYP2B6 | 1 | up |
| CYP2D6 | 1 | up |
| CYP4Z2P | 1 | up |
| DAP | 1 | up |
| DBN1 | 1 | up |
| DCBLD2 | 1 | up |
| DCC | 1 | up |
| DCTD | 1 | up |
| DCTN3 | 1 | up |
| DDX17 | 1 | up |
| DGKH | 1 | up |
| DIAPH2 | 1 | up |
| DISP1 | 1 | up |
| DKFZP586I1420 | 1 | up |
| DKFZp667H046 | 1 | up |
| DLG7 | 1 | up |
| DNAJA1 | 1 | up |
| DNAJB9 | 1 | up |
| DNAJC12 | 1 | up |
| DNAJC9 | 1 | up |
| DNASE1L3 | 1 | up |
| DOPEY2 | 1 | up |
| DPYD | 1 | up |
| DSG1 | 1 | up |
| EFNA1 | 1 | up |
| EFNB3 | 1 | up |
| EGR1 | 1 | up |
| EIF3D | 1 | up |
| ELF2 | 1 | up |
| ELL2 | 1 | up |
| EMD | 1 | up |
| EMG1 | 1 | up |
| EMR3 | 1 | up |
| EPHA2 | 1 | up |
| EPOR | 1 | up |
| ERGIC2 | 1 | up |
| ETS2 | 1 | up |
| EXT2 | 1 | up |
| EZH1 | 1 | up |
| F5 | 1 | up |
| FADD | 1 | up |
| FAM108A1 | 1 | up |
| FAM46A | 1 | up |
| FAU | 1 | up |
| FBL | 1 | up |
| FBN1 | 1 | up |
| FCGR3A | 1 | up |
| FHL3 | 1 | up |
| FLJ20712 | 1 | up |
| FLRT3 | 1 | up |
| FLT1 | 1 | up |
| FNDC1 | 1 | up |
| FTH1 | 1 | up |
| FTHP1 | 1 | up |
| G3BP2 | 1 | up |
| GAGE10 | 1 | up |
| GALNT4 | 1 | up |
| GALT | 1 | up |
| GFRA2 | 1 | up |
| GGT2 | 1 | up |
| GJB2 | 1 | up |
| GK | 1 | up |
| GNAS | 1 | up |
| GPATCH3 | 1 | up |
| GPR1 | 1 | up |
| GPR12 | 1 | up |
| GPR137B | 1 | up |
| GPR171 | 1 | up |
| GPR89A | 1 | up |
| GSPT1 | 1 | up |
| GSTP1 | 1 | up |
| GTF3C5 | 1 | up |
| GUCY2C | 1 | up |
| GZMB | 1 | up |
| H1F0 | 1 | up |
| H6PD | 1 | up |
| HCFC1 | 1 | up |
| HERPUD1 | 1 | up |
| HES1 | 1 | up |
| HGD | 1 | up |
| HHLA1 | 1 | up |
| HIP1 | 1 | up |
| HIP1R | 1 | up |
| HIST1H2AE | 1 | up |
| HLA-A | 1 | up |
| HLA-B | 1 | up |
| HLA-DRB1 | 1 | up |
| HLA-F | 1 | up |
| HMCN1 | 1 | up |
| HMGN2 | 1 | up |
| HMMR | 1 | up |
| HN1L | 1 | up |
| HNRNPA1 | 1 | up |
| HNRNPA2B1 | 1 | up |
| HNRNPA3 | 1 | up |
| HOPX | 1 | up |
| HOXA1 | 1 | up |
| HSP90B1 | 1 | up |
| HYI | 1 | up |
| ICOSLG | 1 | up |
| IER3IP1 | 1 | up |
| IFI44L | 1 | up |
| IGF1 | 1 | up |
| IGF1R | 1 | up |
| IGFBP7 | 1 | up |
| IGL | 1 | up |
| IGSF3 | 1 | up |
| IL1R2 | 1 | up |
| INHBA | 1 | up |
| INHBC | 1 | up |
| INPP4B | 1 | up |
| INSIG1 | 1 | up |
| INSIG2 | 1 | up |
| IPO7 | 1 | up |
| IRF2 | 1 | up |
| ITGA8 | 1 | up |
| ITGB1 | 1 | up |
| ITGB2 | 1 | up |
| ITGB5 | 1 | up |
| ITGB6 | 1 | up |
| ITGBL1 | 1 | up |
| ITLN1 | 1 | up |
| JAK3 | 1 | up |
| KANK1 | 1 | up |
| KATNB1 | 1 | up |
| KCNE4 | 1 | up |
| KCNK4 | 1 | up |
| KDELR3 | 1 | up |
| KIAA0125 | 1 | up |
| KIAA0415 | 1 | up |
| KIAA1107 | 1 | up |
| KIAA1505 | 1 | up |
| KIDINS220 | 1 | up |
| KISS1R | 1 | up |
| KLC1 | 1 | up |
| KLF5 | 1 | up |
| KRT85 | 1 | up |
| KTN1 | 1 | up |
| LAMA2 | 1 | up |
| LAMB3 | 1 | up |
| LARS2 | 1 | up |
| LCN2 | 1 | up |
| LDLRAP1 | 1 | up |
| LEPR | 1 | up |
| LLGL2 | 1 | up |
| LMF1 | 1 | up |
| LMNA | 1 | up |
| LOC100291670 | 1 | up |
| LOC399959 | 1 | up |
| LOC440416 | 1 | up |
| LOC645560 | 1 | up |
| LOC646324 | 1 | up |
| LOC646808 | 1 | up |
| LOC653269 | 1 | up |
| LOC729678 | 1 | up |
| LOC91461 | 1 | up |
| LRRC17 | 1 | up |
| LRRC31 | 1 | up |
| LRRFIP1 | 1 | up |
| LSR | 1 | up |
| LTB4R | 1 | up |
| LTB4R2 | 1 | up |
| LTBP4 | 1 | up |
| LXN | 1 | up |
| LYN | 1 | up |
| MAD2L1 | 1 | up |
| MAD2L1BP | 1 | up |
| MAGI1 | 1 | up |
| MAP7 | 1 | up |
| MAPK14 | 1 | up |
| MAPRE2 | 1 | up |
| MBNL2 | 1 | up |
| MC4R | 1 | up |
| MDK | 1 | up |
| MEA1 | 1 | up |
| MED21 | 1 | up |
| MEF2A | 1 | up |
| METTL9 | 1 | up |
| MFN2 | 1 | up |
| MGATB4 | 1 | up |
| MGST3 | 1 | up |
| MLL | 1 | up |
| MLLT11 | 1 | up |
| MMP12 | 1 | up |
| MORC4 | 1 | up |
| MPG | 1 | up |
| MRPL12 | 1 | up |
| MRPL24 | 1 | up |
| MSH2 | 1 | up |
| MSLN | 1 | up |
| MSR1 | 1 | up |
| MTMR1 | 1 | up |
| MTSS1L | 1 | up |
| MUC4 | 1 | up |
| MYOC | 1 | up |
| MYST3 | 1 | up |
| MYST4 | 1 | up |
| NACA | 1 | up |
| NDEL1 | 1 | up |
| NDRG2 | 1 | up |
| NDUFB4 | 1 | up |
| NDUFS2 | 1 | up |
| NEB | 1 | up |
| NEBL | 1 | up |
| NEDD8 | 1 | up |
| NF2 | 1 | up |
| NFIL3 | 1 | up |
| NGB | 1 | up |
| NGFB | 1 | up |
| NHP2L1 | 1 | up |
| NIT1 | 1 | up |
| NME7 | 1 | up |
| NOTCH3 | 1 | up |
| NPHP1 | 1 | up |
| NPR1 | 1 | up |
| NPYA | 1 | up |
| NQO1 | 1 | up |
| NRG2 | 1 | up |
| NRP2 | 1 | up |
| NTRK2 | 1 | up |
| NUAK1 | 1 | up |
| NUCB2 | 1 | up |
| NUDCD3 | 1 | up |
| OBSL1 | 1 | up |
| OLFML2B | 1 | up |
| OLR1 | 1 | up |
| OPTN | 1 | up |
| ORAI3 | 1 | up |
| OSBPL2 | 1 | up |
| P2RX2 | 1 | up |
| PADI4 | 1 | up |
| PAK1 | 1 | up |
| PANK3 | 1 | up |
| PANK4 | 1 | up |
| PARD6B | 1 | up |
| PBRM1 | 1 | up |
| PCDHB13 | 1 | up |
| PCDHGC3 | 1 | up |
| PCK1 | 1 | up |
| PCM1 | 1 | up |
| PCSK7 | 1 | up |
| PDCL | 1 | up |
| PDGFC | 1 | up |
| PDHB | 1 | up |
| PDLIM5 | 1 | up |
| PDS5B | 1 | up |
| PDZK1IP1 | 1 | up |
| PEX11A | 1 | up |
| PGA5 | 1 | up |
| PHF1 | 1 | up |
| PHF10 | 1 | up |
| PIK3CB | 1 | up |
| PIK3CG | 1 | up |
| PIK3R1 | 1 | up |
| PKP3 | 1 | up |
| PLAT | 1 | up |
| PLEKHO1 | 1 | up |
| PLOD1 | 1 | up |
| PLOD2 | 1 | up |
| PMS2L5 | 1 | up |
| POLD3 | 1 | up |
| POLE2 | 1 | up |
| POLR2H | 1 | up |
| PPAP2A | 1 | up |
| PPL | 1 | up |
| PPP1R10 | 1 | up |
| PPP1R12A | 1 | up |
| PPP2R2A | 1 | up |
| PRAME | 1 | up |
| PRELP | 1 | up |
| PRKAB2 | 1 | up |
| PRKACA | 1 | up |
| PRKACB | 1 | up |
| PRKAG1 | 1 | up |
| PRKAR2A | 1 | up |
| PRMT7 | 1 | up |
| PRSS23 | 1 | up |
| PSAT1 | 1 | up |
| PSMC1 | 1 | up |
| PSMD13 | 1 | up |
| PSMD4 | 1 | up |
| PSMD9 | 1 | up |
| PSME4 | 1 | up |
| PSPHL | 1 | up |
| PTER | 1 | up |
| PTGER2 | 1 | up |
| PTGIS | 1 | up |
| PTMS | 1 | up |
| PTN | 1 | up |
| PTP4A1 | 1 | up |
| PTP4A2 | 1 | up |
| PTPRA | 1 | up |
| PWP1 | 1 | up |
| PYY | 1 | up |
| RAB11FIP5 | 1 | up |
| RABGGTB | 1 | up |
| RAP2A | 1 | up |
| RASSF8 | 1 | up |
| RBM25 | 1 | up |
| RBP1 | 1 | up |
| RELA | 1 | up |
| RNASE2 | 1 | up |
| RNGTT | 1 | up |
| RPL10 | 1 | up |
| RPL17 | 1 | up |
| RPL9 | 1 | up |
| RPN2 | 1 | up |
| RPS4Y1 | 1 | up |
| RUSC1 | 1 | up |
| S100A11 | 1 | up |
| S100A12 | 1 | up |
| S1PR3 | 1 | up |
| SACM1L | 1 | up |
| SAMHD1 | 1 | up |
| SAR1A | 1 | up |
| SCAMP1 | 1 | up |
| SCEL | 1 | up |
| SCOC | 1 | up |
| SCP2 | 1 | up |
| SEC14L2 | 1 | up |
| SEC14L4 | 1 | up |
| SEC62 | 1 | up |
| SERINC1 | 1 | up |
| SERPINB5 | 1 | up |
| SERPINB9 | 1 | up |
| SLC16A10 | 1 | up |
| SLC18A1 | 1 | up |
| SLC22A5 | 1 | up |
| SLC25A13 | 1 | up |
| SLC25A3 | 1 | up |
| SLC25A4 | 1 | up |
| SLC26A4 | 1 | up |
| SLC36A1 | 1 | up |
| SLC44A1 | 1 | up |
| SLC7A5 | 1 | up |
| SLC7A7 | 1 | up |
| SLCO4A1 | 1 | up |
| SLIT2 | 1 | up |
| SMO | 1 | up |
| SMPD4 | 1 | up |
| SNRNP35 | 1 | up |
| SNTA1 | 1 | up |
| SNX4 | 1 | up |
| SOAT1 | 1 | up |
| SORBS1 | 1 | up |
| SORD | 1 | up |
| SOX9 | 1 | up |
| SP110 | 1 | up |
| SPRY4 | 1 | up |
| SSBP3 | 1 | up |
| ST5 | 1 | up |
| ST7OT1 | 1 | up |
| STAMBPL1 | 1 | up |
| STAT5A | 1 | up |
| STCH | 1 | up |
| STIL | 1 | up |
| STK3 | 1 | up |
| STON1 | 1 | up |
| STOX2 | 1 | up |
| STX7 | 1 | up |
| SUB1 | 1 | up |
| SUCLG1 | 1 | up |
| SULT1A3 | 1 | up |
| SULT1C2 | 1 | up |
| SV2A | 1 | up |
| SYN2 | 1 | up |
| TACC2 | 1 | up |
| TAF7 | 1 | up |
| TAS2R13 | 1 | up |
| TAS2R48 | 1 | up |
| TCEA2 | 1 | up |
| TCF12 | 1 | up |
| TESK1 | 1 | up |
| TFAM | 1 | up |
| TFF3 | 1 | up |
| TGFB3 | 1 | up |
| TGFBI | 1 | up |
| TGIF1 | 1 | up |
| THBS2 | 1 | up |
| THBS4 | 1 | up |
| THOC2 | 1 | up |
| THRA | 1 | up |
| THRAP3 | 1 | up |
| TIE1 | 1 | up |
| TIMM17A | 1 | up |
| TIMP3 | 1 | up |
| TLK1 | 1 | up |
| TLOC1 | 1 | up |
| TMEM14A | 1 | up |
| TMEM8A | 1 | up |
| TMEM99 | 1 | up |
| TMSB4X | 1 | up |
| TMX4 | 1 | up |
| TNFRSF11A | 1 | up |
| TNFRSF1A | 1 | up |
| TNFRSF21 | 1 | up |
| TNFRSF6 | 1 | up |
| TNFSF13 | 1 | up |
| TNIK | 1 | up |
| TNPO1 | 1 | up |
| TOX2 | 1 | up |
| TP53BP2 | 1 | up |
| TRAT1 | 1 | up |
| TRIM2 | 1 | up |
| TRIM33 | 1 | up |
| TSC22D1 | 1 | up |
| TSFM | 1 | up |
| TSNARE1 | 1 | up |
| TSPAN3 | 1 | up |
| TTC13 | 1 | up |
| TUBA4A | 1 | up |
| TXNDC1 | 1 | up |
| TXNL2 | 1 | up |
| U2AF2 | 1 | up |
| UACA | 1 | up |
| UBE2E3 | 1 | up |
| UBE2L3 | 1 | up |
| UBXD2 | 1 | up |
| UGGT1 | 1 | up |
| UQCRB | 1 | up |
| UQCRC2 | 1 | up |
| USP19 | 1 | up |
| USP34 | 1 | up |
| USP47 | 1 | up |
| VASP | 1 | up |
| VAT1 | 1 | up |
| VAV1 | 1 | up |
| VCP | 1 | up |
| VKORC1 | 1 | up |
| VPS41 | 1 | up |
| WDHD1 | 1 | up |
| XIST | 1 | up |
| XPNPEP1 | 1 | up |
| YIPF1 | 1 | up |
| YIPF3 | 1 | up |
| YIPF6 | 1 | up |
| YWHAB | 1 | up |
| ZBTB20 | 1 | up |
| ZBTB39 | 1 | up |
| ZDHHC1 | 1 | up |
| ZFHX3 | 1 | up |
| ZFP106 | 1 | up |
| ZNF383 | 1 | up |
| ZNF410 | 1 | up |
| ZNF44 | 1 | up |
| ZNF532 | 1 | up |
| ZNF609 | 1 | up |
| ZNF611 | 1 | up |
| ZNF658 | 1 | up |
| ZNF683 | 1 | up |
| AARS | 1 | down |
| ABCA8 | 1 | down |
| ABCC4 | 1 | down |
| ACBD3 | 1 | down |
| ACOT11 | 1 | down |
| ADAMDEC1 | 1 | down |
| ADAR | 1 | down |
| ADD1 | 1 | down |
| ADH1A | 1 | down |
| ADH1C | 1 | down |
| ADNP2 | 1 | down |
| ADORA2B | 1 | down |
| ADPRH | 1 | down |
| ADRA2A | 1 | down |
| ADRB2 | 1 | down |
| AES | 1 | down |
| AGPAT5 | 1 | down |
| AGRN | 1 | down |
| AIP | 1 | down |
| AKR1A1 | 1 | down |
| AKR1B10 | 1 | down |
| AKR1C3 | 1 | down |
| ALAS1 | 1 | down |
| ALG13 | 1 | down |
| AMOTL2 | 1 | down |
| ANPEP | 1 | down |
| AP2S1 | 1 | down |
| AP4S1 | 1 | down |
| APOBEC3F | 1 | down |
| APOC2 | 1 | down |
| AQP8 | 1 | down |
| ARCN1 | 1 | down |
| ARF1 | 1 | down |
| ARF4 | 1 | down |
| ARF6 | 1 | down |
| ARHT2 | 1 | down |
| ARPC4 | 1 | down |
| ASAHL | 1 | down |
| ASB13 | 1 | down |
| ASPHD2 | 1 | down |
| ASS1 | 1 | down |
| ATG4B | 1 | down |
| ATP1B4 | 1 | down |
| ATP2B1 | 1 | down |
| ATP5D | 1 | down |
| ATP5G1 | 1 | down |
| ATP5J2 | 1 | down |
| ATP6V0B | 1 | down |
| ATP6V1A | 1 | down |
| ATP7A | 1 | down |
| ATXN2 | 1 | down |
| BCAS1 | 1 | down |
| BCKDK | 1 | down |
| BCR | 1 | down |
| BICD2 | 1 | down |
| BMPR1A | 1 | down |
| BPNT1 | 1 | down |
| BRD3 | 1 | down |
| BRI3 | 1 | down |
| BTBD2 | 1 | down |
| BUB3 | 1 | down |
| BZW1 | 1 | down |
| C11orf58 | 1 | down |
| C14orf156 | 1 | down |
| C15orf39 | 1 | down |
| C17orf70 | 1 | down |
| C17orf91 | 1 | down |
| C18orf18 | 1 | down |
| C18orf22 | 1 | down |
| C1orf77 | 1 | down |
| C1QBP | 1 | down |
| C20orf74 | 1 | down |
| C21orf2 | 1 | down |
| C21orf66 | 1 | down |
| C22orf18 | 1 | down |
| C3 | 1 | down |
| C3orf34 | 1 | down |
| C4BPA | 1 | down |
| C4orf19 | 1 | down |
| C4orf38 | 1 | down |
| C6orf105 | 1 | down |
| C6orf62 | 1 | down |
| C9orf53 | 1 | down |
| CA12 | 1 | down |
| CA3 | 1 | down |
| CA4 | 1 | down |
| CABC1 | 1 | down |
| CABIN1 | 1 | down |
| CALR | 1 | down |
| CALU | 1 | down |
| CAMK2B | 1 | down |
| CAMTA2 | 1 | down |
| CANX | 1 | down |
| CAP2 | 1 | down |
| CAPG | 1 | down |
| CAPN10 | 1 | down |
| CAPZA2 | 1 | down |
| CASKIN2 | 1 | down |
| CASP6 | 1 | down |
| CBS | 1 | down |
| CCBL1 | 1 | down |
| CCL11 | 1 | down |
| CCL19 | 1 | down |
| CCL24 | 1 | down |
| CCL28 | 1 | down |
| CCNB1 | 1 | down |
| CCNB2 | 1 | down |
| CCT5 | 1 | down |
| CCT7 | 1 | down |
| CD164 | 1 | down |
| CD1C | 1 | down |
| CD24 | 1 | down |
| CD2AP | 1 | down |
| CD38 | 1 | down |
| CD52 | 1 | down |
| CD79A | 1 | down |
| CDC42SE1 | 1 | down |
| CDC45L | 1 | down |
| CDCA3 | 1 | down |
| CDH11 | 1 | down |
| CDH17 | 1 | down |
| CDH8 | 1 | down |
| CH25H | 1 | down |
| CHCHD2 | 1 | down |
| CHN1 | 1 | down |
| CHP2 | 1 | down |
| CHST2 | 1 | down |
| CIRBP | 1 | down |
| CITED2 | 1 | down |
| CKB | 1 | down |
| CKMT2 | 1 | down |
| CLDN8 | 1 | down |
| CLEC4M | 1 | down |
| CLIC1 | 1 | down |
| CLTC | 1 | down |
| CNN3 | 1 | down |
| CNOT7 | 1 | down |
| CNPY3 | 1 | down |
| CNTN1 | 1 | down |
| COG5 | 1 | down |
| COIL | 1 | down |
| COL6A2 | 1 | down |
| COLEC10 | 1 | down |
| COMT | 1 | down |
| COPB1 | 1 | down |
| COPZ2 | 1 | down |
| COX6B2 | 1 | down |
| COX7A2 | 1 | down |
| COX7A2L | 1 | down |
| COX7C | 1 | down |
| CPSF1 | 1 | down |
| CRABP1 | 1 | down |
| CRKL | 1 | down |
| CSE1L | 1 | down |
| CSN3 | 1 | down |
| CSNK1D | 1 | down |
| CSNK1G2 | 1 | down |
| CSNK2A1 | 1 | down |
| CTSA | 1 | down |
| CTSS | 1 | down |
| CXCL13 | 1 | down |
| CYB5B | 1 | down |
| CYCS | 1 | down |
| CYP19A1 | 1 | down |
| CYP2A13 | 1 | down |
| D4S234E | 1 | down |
| DAD1 | 1 | down |
| DBF4B | 1 | down |
| DCN | 1 | down |
| DDR2 | 1 | down |
| DDX18 | 1 | down |
| DDX3X | 1 | down |
| DDX3Y | 1 | down |
| DDX52 | 1 | down |
| DDX59 | 1 | down |
| DENND2A | 1 | down |
| DIO2 | 1 | down |
| DKFZP564O0823 | 1 | down |
| DLAT | 1 | down |
| DLG3 | 1 | down |
| DLG4 | 1 | down |
| DLST | 1 | down |
| DMWD | 1 | down |
| DNAJC10 | 1 | down |
| DNTTIP2 | 1 | down |
| DOCK6 | 1 | down |
| DSC2 | 1 | down |
| DTYMK | 1 | down |
| DUOX2 | 1 | down |
| DUSP1 | 1 | down |
| DUSP6 | 1 | down |
| DVL1 | 1 | down |
| DYNLL1 | 1 | down |
| DYNLT1 | 1 | down |
| DYRK2 | 1 | down |
| EDAR | 1 | down |
| EEF1B2 | 1 | down |
| EGF | 1 | down |
| EGFR | 1 | down |
| EIF1 | 1 | down |
| EIF2B3 | 1 | down |
| EIF2S2 | 1 | down |
| EIF3CL | 1 | down |
| EIF3I | 1 | down |
| EIF4A3 | 1 | down |
| EIF5A | 1 | down |
| ELAC1 | 1 | down |
| ELK4 | 1 | down |
| ELOVL4 | 1 | down |
| ENG | 1 | down |
| ENO1 | 1 | down |
| ENTPD5 | 1 | down |
| ENTPD6 | 1 | down |
| EPB41L4B | 1 | down |
| EPM2A | 1 | down |
| EPPK1 | 1 | down |
| ERBB2 | 1 | down |
| ERBB4 | 1 | down |
| ERCC4 | 1 | down |
| ESD | 1 | down |
| ESM1 | 1 | down |
| ETFA | 1 | down |
| ETS1 | 1 | down |
| FABP2 | 1 | down |
| FAM172A | 1 | down |
| FAM3A | 1 | down |
| FAM84A | 1 | down |
| FGB | 1 | down |
| FILIP1L | 1 | down |
| FLT3 | 1 | down |
| FNDC3B | 1 | down |
| FOXJ3 | 1 | down |
| FOXO3 | 1 | down |
| FOXO4 | 1 | down |
| FTCD | 1 | down |
| FTSJ3 | 1 | down |
| FUSIP1 | 1 | down |
| FXR2 | 1 | down |
| FXYD3 | 1 | down |
| FYN | 1 | down |
| GAB3 | 1 | down |
| GALNACT-2 | 1 | down |
| GALNT3 | 1 | down |
| GAS6 | 1 | down |
| GAS7 | 1 | down |
| GATA3 | 1 | down |
| GATA6 | 1 | down |
| GCAT | 1 | down |
| GFER | 1 | down |
| GNAI3 | 1 | down |
| GNB1 | 1 | down |
| GNB1L | 1 | down |
| GNG12 | 1 | down |
| GORASP2 | 1 | down |
| GPC2 | 1 | down |
| GPSM3 | 1 | down |
| GRK6 | 1 | down |
| GRSF1 | 1 | down |
| GRTP1 | 1 | down |
| GSTM1 | 1 | down |
| GSTM3 | 1 | down |
| GTF2F1 | 1 | down |
| GTF2H4 | 1 | down |
| GUCA2B | 1 | down |
| H4PG | 1 | down |
| HAUS8 | 1 | down |
| HELZ | 1 | down |
| HERC4 | 1 | down |
| HIF1A | 1 | down |
| HIPK1 | 1 | down |
| HIPK3 | 1 | down |
| HIST1H4C | 1 | down |
| HLA-C | 1 | down |
| HLA-DQB1 | 1 | down |
| HMGA2 | 1 | down |
| HMGB2 | 1 | down |
| HNRNPF | 1 | down |
| HNRPC | 1 | down |
| HPN | 1 | down |
| HPSE | 1 | down |
| HS2ST1 | 1 | down |
| HS3ST5 | 1 | down |
| HSB3B2 | 1 | down |
| HSD11B2 | 1 | down |
| HSD17B2 | 1 | down |
| HSD17B8 | 1 | down |
| HSP90AA1 | 1 | down |
| HSPA1A | 1 | down |
| HSPA5 | 1 | down |
| HSPA8 | 1 | down |
| HSPA9 | 1 | down |
| ICAM1 | 1 | down |
| ICAM2 | 1 | down |
| ID1 | 1 | down |
| ID4 | 1 | down |
| IDH1 | 1 | down |
| IDH2 | 1 | down |
| IFNAR1 | 1 | down |
| IFNGR2 | 1 | down |
| IGFBP1 | 1 | down |
| IGFBP5 | 1 | down |
| IGHG1 | 1 | down |
| IGHM | 1 | down |
| IGJ | 1 | down |
| IGKC | 1 | down |
| IGKV4-1 | 1 | down |
| IGL@ | 1 | down |
| IGLJ3 | 1 | down |
| IKBKG | 1 | down |
| IL12RB1 | 1 | down |
| IL17RA | 1 | down |
| IL17RC | 1 | down |
| IL27RA | 1 | down |
| IL28RA | 1 | down |
| INCENP | 1 | down |
| INTS1 | 1 | down |
| IRAK1BP1 | 1 | down |
| IRF1 | 1 | down |
| ITGA2 | 1 | down |
| ITGA6 | 1 | down |
| ITIH1 | 1 | down |
| JUN | 1 | down |
| JUND | 1 | down |
| KBTBD11 | 1 | down |
| KCNK3 | 1 | down |
| KCNQ4 | 1 | down |
| KHSRP | 1 | down |
| KIAA0467 | 1 | down |
| KIAA0828 | 1 | down |
| KIAA1919 | 1 | down |
| KIT | 1 | down |
| KLF13 | 1 | down |
| KLF7 | 1 | down |
| KLHL22 | 1 | down |
| KLRB1 | 1 | down |
| KPNA1 | 1 | down |
| KPNA2 | 1 | down |
| KRT23 | 1 | down |
| LAGE3 | 1 | down |
| LAMA3 | 1 | down |
| LAMB1 | 1 | down |
| LAMP1 | 1 | down |
| LAP3 | 1 | down |
| LARP1 | 1 | down |
| LARP4 | 1 | down |
| LAT | 1 | down |
| LGALS2 | 1 | down |
| LIF | 1 | down |
| LILRB3 | 1 | down |
| LMNB1 | 1 | down |
| LOC100130100 | 1 | down |
| LOC100133109 | 1 | down |
| LOC282997 | 1 | down |
| LOC401068 | 1 | down |
| LOC552889 | 1 | down |
| LOH11CR2A | 1 | down |
| LRIG1 | 1 | down |
| LRP1 | 1 | down |
| LRPPRC | 1 | down |
| LRRC34 | 1 | down |
| LRRC37B2 | 1 | down |
| LRRC41 | 1 | down |
| LRRC59 | 1 | down |
| LSAMP | 1 | down |
| LSM3 | 1 | down |
| LSM4 | 1 | down |
| MAFG | 1 | down |
| MALAT-1 | 1 | down |
| MAN2A2 | 1 | down |
| MAP2K1 | 1 | down |
| MAP2K6 | 1 | down |
| MAP3K4 | 1 | down |
| MAP3K8 | 1 | down |
| MAPK1 | 1 | down |
| MAPK13 | 1 | down |
| MAPRE1 | 1 | down |
| MARCH3 | 1 | down |
| MARS | 1 | down |
| MBD2 | 1 | down |
| MCL1 | 1 | down |
| MDH1 | 1 | down |
| ME2 | 1 | down |
| MEP1A | 1 | down |
| METTL7A | 1 | down |
| MEX3C | 1 | down |
| MFAP1 | 1 | down |
| MGAT5 | 1 | down |
| MKI67IP | 1 | down |
| MMADHC | 1 | down |
| MMP1 | 1 | down |
| MMP13 | 1 | down |
| MMP2 | 1 | down |
| MNT | 1 | down |
| MOBKL2B | 1 | down |
| MORF4L2 | 1 | down |
| MPDU1 | 1 | down |
| MRCL3 | 1 | down |
| MRPL16 | 1 | down |
| MRPL40 | 1 | down |
| MRPS11 | 1 | down |
| MRPS23 | 1 | down |
| MS4A1 | 1 | down |
| MT1A | 1 | down |
| MT1G | 1 | down |
| MT1H | 1 | down |
| MTF2 | 1 | down |
| MTFR1 | 1 | down |
| MTO1 | 1 | down |
| MUM1L1 | 1 | down |
| MYCBP | 1 | down |
| MYCBP2 | 1 | down |
| MYF6 | 1 | down |
| MYH3 | 1 | down |
| MYL1 | 1 | down |
| MYOF | 1 | down |
| MYOM1 | 1 | down |
| MYOT | 1 | down |
| N4BP2L2 | 1 | down |
| NAA10 | 1 | down |
| NAA15 | 1 | down |
| NAP1L4 | 1 | down |
| NARS | 1 | down |
| NAT1 | 1 | down |
| NCOA1 | 1 | down |
| NDFIP1 | 1 | down |
| NDUFA4 | 1 | down |
| NDUFA6 | 1 | down |
| NDUFA9 | 1 | down |
| NDUFV1 | 1 | down |
| NEK6 | 1 | down |
| NELF | 1 | down |
| NET1 | 1 | down |
| NFE2L2 | 1 | down |
| NFKBIZ | 1 | down |
| NFYC | 1 | down |
| NIPA1 | 1 | down |
| NISCH | 1 | down |
| NME2 | 1 | down |
| NMNAT3 | 1 | down |
| NOLC1 | 1 | down |
| NOMO1 | 1 | down |
| NOP16 | 1 | down |
| NOTCH2 | 1 | down |
| NP | 1 | down |
| NPEPPS | 1 | down |
| NPM1 | 1 | down |
| NR0B1 | 1 | down |
| NR1D2 | 1 | down |
| NR1H4 | 1 | down |
| NR5A2 | 1 | down |
| NRARP | 1 | down |
| NRBP1 | 1 | down |
| NTN3 | 1 | down |
| NUCB1 | 1 | down |
| NUFIP1 | 1 | down |
| NUP155 | 1 | down |
| NUS1 | 1 | down |
| OBP2B | 1 | down |
| OGT | 1 | down |
| OPHN1 | 1 | down |
| P2RY14 | 1 | down |
| P4HA1 | 1 | down |
| P4HB | 1 | down |
| PADI2 | 1 | down |
| PAPPA | 1 | down |
| PARVB | 1 | down |
| PCMT1 | 1 | down |
| PCNA | 1 | down |
| PDAP1 | 1 | down |
| PDCD11 | 1 | down |
| PDCD4 | 1 | down |
| PDCD5 | 1 | down |
| PDE9A | 1 | down |
| PDIA6 | 1 | down |
| PDIA6 | 1 | down |
| PDPK1 | 1 | down |
| PDPN | 1 | down |
| PFDN2 | 1 | down |
| PFKL | 1 | down |
| PGK1 | 1 | down |
| PGPEP1 | 1 | down |
| PHLDA1 | 1 | down |
| PIGR | 1 | down |
| PIK3CA | 1 | down |
| PKIA | 1 | down |
| PKM2 | 1 | down |
| PLA2G10 | 1 | down |
| PLA2G2A | 1 | down |
| PLAUR | 1 | down |
| PLCE1 | 1 | down |
| PLEKHB2 | 1 | down |
| PLK1 | 1 | down |
| PLXNB2 | 1 | down |
| PMF1 | 1 | down |
| PMS1 | 1 | down |
| POLD2 | 1 | down |
| POLR2I | 1 | down |
| POMP | 1 | down |
| PON2 | 1 | down |
| POU2AF1 | 1 | down |
| PPARG | 1 | down |
| PPIB | 1 | down |
| PPID | 1 | down |
| PPM1G | 1 | down |
| PPP1R1C | 1 | down |
| PPP2CA | 1 | down |
| PPP2R1A | 1 | down |
| PRDX1 | 1 | down |
| PRDX2 | 1 | down |
| PRELID1 | 1 | down |
| PRKAB1 | 1 | down |
| PRKAR1B | 1 | down |
| PRKD1 | 1 | down |
| PRMT1 | 1 | down |
| PRPF40A | 1 | down |
| PRTN3 | 1 | down |
| PSD4 | 1 | down |
| PSG11 | 1 | down |
| PSG9 | 1 | down |
| PSMA4 | 1 | down |
| PSMB1 | 1 | down |
| PSMB10 | 1 | down |
| PSMB2 | 1 | down |
| PSMB4 | 1 | down |
| PSMB8 | 1 | down |
| PSMB9 | 1 | down |
| PSMC4 | 1 | down |
| PSMD14 | 1 | down |
| PSMD8 | 1 | down |
| PSME1 | 1 | down |
| PSME2 | 1 | down |
| PSME3 | 1 | down |
| PTGES3 | 1 | down |
| PTGS2 | 1 | down |
| PTK2B | 1 | down |
| PTPN11 | 1 | down |
| PTPN13 | 1 | down |
| PTPRC | 1 | down |
| PTPRD | 1 | down |
| PTPRN2 | 1 | down |
| PYCR1 | 1 | down |
| R3HDML | 1 | down |
| RAB11FIP4 | 1 | down |
| RAB28 | 1 | down |
| RAI14 | 1 | down |
| RAN | 1 | down |
| RANBP2 | 1 | down |
| RAPGEF1 | 1 | down |
| RASGRP1 | 1 | down |
| RCAN1 | 1 | down |
| RCC1 | 1 | down |
| RCOR1 | 1 | down |
| RDX | 1 | down |
| REG1A | 1 | down |
| REG3A | 1 | down |
| REST | 1 | down |
| RFWD3 | 1 | down |
| RFXANK | 1 | down |
| RGMB | 1 | down |
| RGS5 | 1 | down |
| RGS9 | 1 | down |
| RHBDD3 | 1 | down |
| RHEB | 1 | down |
| RHOA | 1 | down |
| RNF181 | 1 | down |
| RNF213 | 1 | down |
| RP11-298P3.3 | 1 | down |
| RPA3 | 1 | down |
| RPL15 | 1 | down |
| RPL27A | 1 | down |
| RPL29 | 1 | down |
| RPL37A | 1 | down |
| RPL38 | 1 | down |
| RPL39 | 1 | down |
| RPL5 | 1 | down |
| RPL6 | 1 | down |
| RPLP2 | 1 | down |
| RPS11 | 1 | down |
| RPS14 | 1 | down |
| RPS19 | 1 | down |
| RPS21 | 1 | down |
| RPS23 | 1 | down |
| RPS27L | 1 | down |
| RPS28 | 1 | down |
| RPS3A | 1 | down |
| RPS6 | 1 | down |
| RPS7 | 1 | down |
| Rqcd1 | 1 | down |
| RRM1 | 1 | down |
| RRM2 | 1 | down |
| RSAD2 | 1 | down |
| RSL24D1 | 1 | down |
| RUNX1 | 1 | down |
| RYR3 | 1 | down |
| S100A10 | 1 | down |
| S100A3 | 1 | down |
| SAT1 | 1 | down |
| SBF1 | 1 | down |
| SBK1 | 1 | down |
| SCO1 | 1 | down |
| SDCBP | 1 | down |
| SDF4 | 1 | down |
| SEC11A | 1 | down |
| SEC24D | 1 | down |
| SELT | 1 | down |
| SEPT_2 | 1 | down |
| SET | 1 | down |
| SFN | 1 | down |
| SFRS17A | 1 | down |
| SFRS3 | 1 | down |
| SFRS9 | 1 | down |
| SGPP2 | 1 | down |
| SI | 1 | down |
| SILV | 1 | down |
| SLAIN2 | 1 | down |
| SLC17A7 | 1 | down |
| SLC25A16 | 1 | down |
| SLC25A17 | 1 | down |
| SLC25A30 | 1 | down |
| SLC25A37 | 1 | down |
| SLC26A2 | 1 | down |
| SLC26A3 | 1 | down |
| SLC2A5 | 1 | down |
| SLC35B1 | 1 | down |
| SLC35E3 | 1 | down |
| SLC39A6 | 1 | down |
| SLC6A13 | 1 | down |
| SLPI | 1 | down |
| SMAD1 | 1 | down |
| SMAD7 | 1 | down |
| SNCA | 1 | down |
| SNCAIP | 1 | down |
| SNRPB2 | 1 | down |
| SNRPD2 | 1 | down |
| SNX26 | 1 | down |
| SNX7 | 1 | down |
| SON | 1 | down |
| SORBS2 | 1 | down |
| SPCS1 | 1 | down |
| SPDYA | 1 | down |
| SPEN | 1 | down |
| SPG7 | 1 | down |
| SPOP | 1 | down |
| SPPL2B | 1 | down |
| SPTAN1 | 1 | down |
| SRP19 | 1 | down |
| SRPR | 1 | down |
| SSR1 | 1 | down |
| SSR2 | 1 | down |
| SSRP1 | 1 | down |
| ST6GAL2 | 1 | down |
| STAP2 | 1 | down |
| STAT2 | 1 | down |
| STC1 | 1 | down |
| STK17A | 1 | down |
| STK25 | 1 | down |
| STRAP | 1 | down |
| SULT1B1 | 1 | down |
| SUMO2 | 1 | down |
| SUPTH5 | 1 | down |
| SYPL1 | 1 | down |
| SYT13 | 1 | down |
| SYT17 | 1 | down |
| TACSTD1 | 1 | down |
| TAF12 | 1 | down |
| TBC1D2B | 1 | down |
| TBCA | 1 | down |
| TBCB | 1 | down |
| TBL2 | 1 | down |
| TCEB2 | 1 | down |
| TCIRG1 | 1 | down |
| TCP1 | 1 | down |
| TETRAN | 1 | down |
| TEX11 | 1 | down |
| TFG | 1 | down |
| TFRC | 1 | down |
| TGFBR2 | 1 | down |
| THOC4 | 1 | down |
| THOC5 | 1 | down |
| THPO | 1 | down |
| TIMM23 | 1 | down |
| TLE1 | 1 | down |
| TLE6 | 1 | down |
| TM2D1 | 1 | down |
| TM4SF5 | 1 | down |
| TMBIM6 | 1 | down |
| TMED10 | 1 | down |
| TMED2 | 1 | down |
| TMED5 | 1 | down |
| TMEM14C | 1 | down |
| TMEM184B | 1 | down |
| TMEM46 | 1 | down |
| TMEM49 | 1 | down |
| TMEM97 | 1 | down |
| TMEM98 | 1 | down |
| TNFRSF10B | 1 | down |
| TNFRSF17 | 1 | down |
| TNFRSF1B | 1 | down |
| TNFSF11 | 1 | down |
| TNNC1 | 1 | down |
| TNRC6B | 1 | down |
| TPM1 | 1 | down |
| TPM3 | 1 | down |
| TPM4 | 1 | down |
| TPP2 | 1 | down |
| TRA2B | 1 | down |
| TRAF3 | 1 | down |
| TRAF4 | 1 | down |
| TRIM15 | 1 | down |
| TRIM36 | 1 | down |
| TRPV4 | 1 | down |
| TSPAN1 | 1 | down |
| TSPAN7 | 1 | down |
| TUBA1B | 1 | down |
| TUBB | 1 | down |
| TUBB2B | 1 | down |
| TUBG1 | 1 | down |
| TXNL1 | 1 | down |
| TXNL4A | 1 | down |
| TYK2 | 1 | down |
| UBE2D3 | 1 | down |
| UBE2G2 | 1 | down |
| UBE2T | 1 | down |
| UBE2V2 | 1 | down |
| UBE2Z | 1 | down |
| UBE4A | 1 | down |
| UCHL3 | 1 | down |
| UCP2 | 1 | down |
| UGDH | 1 | down |
| UGT1A6 | 1 | down |
| UGT2B15 | 1 | down |
| UGT2B17 | 1 | down |
| UNC45A | 1 | down |
| UQCR | 1 | down |
| USP1 | 1 | down |
| USP9X | 1 | down |
| UTP18 | 1 | down |
| UTP6 | 1 | down |
| VDAC3 | 1 | down |
| VHL | 1 | down |
| VILL | 1 | down |
| VIP | 1 | down |
| VPS37C | 1 | down |
| VPS4B | 1 | down |
| WASL | 1 | down |
| WDR61 | 1 | down |
| WNT5A | 1 | down |
| XRCC6 | 1 | down |
| YME1L1 | 1 | down |
| YWHAG | 1 | down |
| YWHAZ | 1 | down |
| ZBTB16 | 1 | down |
| ZC3H7B | 1 | down |
| ZFP36L1 | 1 | down |
| ZFY | 1 | down |
| ZG16 | 1 | down |
| ZIC4 | 1 | down |
| ZNF148 | 1 | down |
| ZNF207 | 1 | down |
| ZNF37A | 1 | down |
| ZNF587 | 1 | down |
| ZNF721 | 1 | down |
| ZSCAN21 | 1 | down |
| RBM6 | 1 | both |
