# Supplementary material for: Consensus Pathways Implicated in Prognosis of Colorectal Cancer Identified Through Systematic Enrichment Analysis of Gene Expression Profiling Studies
Source: PLoS One. 2011 Apr 25;6(4):e18867. doi: 10.1371/journal.pone.0018867 (PMC3081819; doi:10.1371/journal.pone.0018867)
Supplement: Table S3 — Results of all enrichment tools used with the 54 gene list. Only those categories selected by at least two enrichment tools are shown. In each case, the first row represents the overrepresentation P value adjusted for multiple testing, and the second row the number of genes in the category within the 54 gene list. Table S3A. Results for Gene Ontology Biological Process categories; Table S3B. Results for Gene Ontology Molecular Function categories; Table S3C. Results for KEGG pathway categories. (DOC) [file pone.0018867.s005.doc]

**Table S3. Results of all enrichment tools used with the 54 gene list.**

Only those categories selected by at least two enrichment tools are shown. In each case, the first row represents the overrepresentation P value adjusted for multiple testing, and the second row the number of genes in the category within the 54 gene list.

**Table S3A.** Results for Gene Ontology Biological Process categories.

| **ID** | **Category** | **GOTM** | **GATHER** | **WebGestalt** | **ToppFun** | **FatiGO** | **g:Profiler** | **DAVID** | **GeneCodis** |
| --- | --- | --- | --- | --- | --- | --- | --- | --- | --- |
| **Total number of significant categories** | | **10** | **0** | **40** | **35** | **1** | **9** | **0** | **26** |
| GO:0006915 | apoptosis |  |  | 7.90E-03  12 | 2.70E-05  14 |  |  |  | 2.77E-02  3 |
| GO:0031103 | axon regeneration |  |  | 2.03E-02  2 | 1.51E-03  2 |  |  |  |  |
| GO:0008219 | cell death |  |  | 1.68E-02  12 | 7.30E-05  14 |  |  |  |  |
| GO:0031958 | corticosteroid receptor signaling pathway |  |  | 7.90E-03  2 | 4.07E-04  2 |  |  |  |  |
| GO:0016265 | death |  |  | 1.68E-02  12 | 7.60E-05  14 | 1.04E-02  10 |  |  |  |
| GO:0046697 | decidualization |  |  | 1.85E-02  2 | 1.02E-03  2 |  |  |  |  |
| GO:0042921 | glucocorticoid receptor signaling pathway |  |  | 6.50E-03  2 | 3.17E-04  2 |  |  |  | 2.64E-03  2 |
| GO:0060333 | interferon-gamma-mediated signaling pathway | 3.50E-03  2 |  | 3.20E-03  2 | 6.90E-05  2 |  |  |  |  |
| GO:0051452 | intracellular pH reduction |  |  | 2.10E-02  2 | 1.51E-03  2 |  |  |  |  |
| GO:0001893 | maternal placenta development |  |  | 2.03E-02  2 | 1.51E-03  2 |  |  |  |  |
| GO:0048519 | negative regulation of biological process | 3.70E-03  17 |  | 2.21E-02  14 |  |  | 2.00E-05  16 |  |  |
| GO:0031330 | negative regulation of cellular catabolic process |  |  | 3.20E-03  3 | 4.80E-05  3 |  |  |  |  |
| GO:0048523 | negative regulation of cellular process | 2.10E-03  16 |  | 1.80E-02  14 |  |  | 6.09E-06  16 |  |  |
| GO:0048518 | positive regulation of biological process | 2.00E-03  18 |  | 6.50E-03  17 |  |  | 3.18E-06  18 |  |  |
| GO:0045780 | positive regulation of bone resorption |  |  | 3.20E-03  2 | 1.14E-04  2 |  |  |  |  |
| GO:0048522 | positive regulation of cellular process | 1.80E-03  18 |  | 3.20E-03  17 |  |  | 6.56E-07  18 |  |  |
| GO:0012501 | programmed cell death | 3.70E-03  12 |  | 7.90E-03  12 | 3.00E-05  14 |  |  |  |  |
| GO:0042981 | regulation of apoptosis |  |  |  | 8.48E-04  10 |  |  |  | 2.33E-02  2 |
| GO:0045124 | regulation of bone resorption |  |  | 1.85E-02  2 | 1.34E-03  2 |  |  |  |  |
| GO:0031329 | regulation of cellular catabolic process |  |  | 2.10E-02  3 | 1.30E-03  3 |  |  |  |  |
| GO:0001959 | regulation of cytokine-mediated signaling pathway |  |  | 1.85E-02  2 | 1.17E-03  2 |  |  |  |  |
| GO:0060334 | regulation of interferon-gamma-mediated signaling pathway | 2.10E-03  2 |  | 3.20E-03  2 | 3.40E-05  2 |  | 2.27E-05  2 |  | 1.89E-04  2 |
| GO:0060338 | regulation of type I interferon-mediated signaling pathway | 2.00E-03  2 |  | 3.20E-03  2 | 1.10E-05  2 |  | 7.57E-06  2 |  | 1.89E-04  2 |
| GO:0010033 | response to organic substance |  |  | 1.64E-02  9 | 5.05E-04  7 |  | 9.55E-06  11 |  |  |
| GO:0051789 | response to protein stimulus | 2.00E-03  6 |  | 3.20E-03  5 | 4.00E-06  6 |  | 6.29E-06  6 |  |  |
| GO:0006986 | response to unfolded protein |  |  | 3.20E-03  4 | 1.44E-04  4 |  |  |  | 2.93E-02  2 |
| GO:0033280 | response to vitamin D |  |  | 2.10E-02  2 | 1.34E-03  2 |  |  |  |  |
| GO:0060337 | type I interferon-mediated signaling pathway | 2.10E-03  2 |  | 3.20E-03  2 | 3.40E-05  2 |  |  |  |  |
| Number of significant categories only with this tool | | 0 | 0 | 13 | 1 | 0 | 0 | 0 | 20 |

**Table S3B.** Results for Gene Ontology Molecular Function categories.

| **ID** | **Category** | **GOTM** | **WebGestalt** | **ToppFun** | **FatiGO** | **g:Profiler** | **DAVID** | **GeneCodis** |
| --- | --- | --- | --- | --- | --- | --- | --- | --- |
| **Total number of significant categories** | | **10** | **12** | **3** | **0** | **0** | **0** | **17** |
| GO:0051087 | chaperone binding |  | 3.02E-02  2 |  |  |  |  | 7.28E-03  2 |
| GO:0005518 | collagen binding |  | 4.85E-02  2 |  |  |  |  | 8.85E-03  2 |
| GO:0004857 | enzyme inhibitor activity | 1.29E-02  6 | 3.02E-02  5 |  |  |  |  |  |
| GO:0030234 | enzyme regulator activity | 1.29E-02  10 | 3.02E-02  9 | 4.03E-04  10 |  |  |  |  |
| GO:0050998 | nitric-oxide synthase binding | 1.11E-02  2 |  | 2.74E-04  2 |  |  |  | 1.81E-03  2 |
| GO:0015459 | potassium channel regulator activity | 2.81E-02  2 | 3.02E-02  2 |  |  |  |  |  |
| GO:0005515 | protein binding | 1.11E-02  39 | 3.02E-02  38 |  |  |  |  | 1.06E-05  23 |
| GO:0046961 | proton-transporting ATPase activity, rotational mechanism | 2.34E-02  2 | 3.02E-02  2 |  |  |  |  | 7.28E-03  2 |
| GO:0051082 | unfolded protein binding | 1.29E-02  4 | 3.02E-02  4 | 4.82E-04  4 |  |  |  | 8.18E-04  4 |
| Number of significant categories only with this tool | | 3 | 4 | 0 | 0 | 0 | 0 | 11 |

**Table S3C.** Results for KEGG pathway categories.

| **ID** | **Category** | **GATHER** | **WebGestalt** | **ConsensusPathDB** | **ToppFun** | **g:Profiler** | **DAVID** | **GeneCodis** |
| --- | --- | --- | --- | --- | --- | --- | --- | --- |
| **Total number of significant categories** | | **0** | **13** | **2** | **0** | **0** | **0** | **8** |
| KEGG4520 | Adherens junction |  | 6.20E-03  2 |  |  |  |  | 4.50E-03  2 |
| KEGG4512 | ECM-receptor interaction |  | 6.60E-03  2 |  |  |  |  | 5.64E-03  2 |
| KEGG4510 | Focal adhesion |  | 5.40E-03  3 |  |  |  |  | 2.38E-03  3 |
| KEGG4115 | p53 signaling pathway |  | 5.80E-03  2 |  |  |  |  | 3.80E-03  2 |
| KEGG5200 | Pathways in cancer |  | 5.40E-03  4 |  |  |  |  | 9.54E-04  4 |
| KEGG4810 | Regulation of actin cytoskeleton |  | 5.40E-03  3 |  |  |  |  | 2.82E-03  3 |
| KEGG4940 | Type I diabetes mellitus |  | 5.40E-03  2 | 1.76E-02  2 |  |  |  | 1.51E-03  2 |
| KEGG5110 | Vibrio cholerae infection |  | 5.40E-03  2 | 2.16E-02  2 |  |  |  | 2.86E-03  2 |
| Number of significant categories only with this tool | | 0 | 5 | 0 | 0 | 0 | 0 | 0 |
