# Supplementary material for: Consensus Pathways Implicated in Prognosis of Colorectal Cancer Identified Through Systematic Enrichment Analysis of Gene Expression Profiling Studies
Source: PLoS One. 2011 Apr 25;6(4):e18867. doi: 10.1371/journal.pone.0018867 (PMC3081819; doi:10.1371/journal.pone.0018867)
Supplement: Table S4 — Results of all enrichment tools used with the 124 gene list. Only those categories selected by at least two enrichment tools are shown. In each case, the first row represents the overrepresentation P value adjusted for multiple testing, and the second row the number of genes in the category within the 124 gene list. Table S4A. Results for Gene Ontology Biological Process categories; Table S4B. Results for Gene Ontology Molecular Function categories; Table S4C. Results for KEGG pathway categories. (DOC) [file pone.0018867.s006.doc]

**Table S4. Results of all enrichment tools used with the 124 gene list.**

Only those categories selected by at least two enrichment tools are shown. In each case, the first row represents the overrepresentation P value adjusted for multiple testing, and the second row the number of genes in the category within the 124 gene list.

**Table S4A.** Results for Gene Ontology Biological Process categories.

| **ID** | **Category** | **GOTM** | **GATHER** | **WebGestalt** | **ToppFun** | **FatiGO** | **g:Profiler** | **DAVID** | **GeneCodis** |
| --- | --- | --- | --- | --- | --- | --- | --- | --- | --- |
| **Total number of significant categories** | | **10** | **1** | **40** | **29** | **0** | **16** | **0** | **54** |
| GO:0048856 | anatomical structure development |  |  | 3.38E-02  34 |  |  | 1.55E-06  36 |  |  |
| GO:0006915 | apoptosis | 2.50E-03  21 |  | 1.88E-02  20 | 3.00E-05  23 |  |  |  | 4.96E-02  4 |
| GO:0002326 | B cell lineage commitment |  |  | 2.20E-02  2 | 5.88E-04  2 |  |  |  | 5.44E-03  2 |
| GO:0008219 | cell death | 2.50E-03  22 |  | 2.08E-02  21 | 4.20E-05  24 |  |  |  |  |
| GO:0008283 | cell proliferation | 2.40E-03  22 |  | 1.16E-02  22 | 1.47E-04  22 |  |  |  | 2.22E-02  5 |
| GO:0016265 | death | 2.50E-03  22 |  | 2.08E-02  21 | 4.50E-05  24 |  |  |  |  |
| GO:0046697 | decidualization |  |  | 1.32E-02  3 | 1.56E-04  3 |  |  |  |  |
| GO:0042921 | glucocorticoid receptor signaling pathway |  |  | 2.53E-02  2 |  |  |  |  | 8.53E-03  2 |
| GO:0060333 | interferon-gamma-mediated signaling pathway |  |  | 1.88E-02  2 | 3.55E-04  2 |  |  |  |  |
| GO:0001893 | maternal placenta development |  |  | 1.46E-02  3 | 2.86E-04  3 |  |  |  |  |
| GO:0008285 | negative regulation of cell proliferation |  |  | 3.38E-02  9 | 8.81E-04  10 |  |  |  | 4.49E-03  6 |
| GO:0031330 | negative regulation of cellular catabolic process |  |  | 2.08E-02  3 | 5.48E-04  3 |  |  |  |  |
| GO:0048518 | positive regulation of biological process | 2.20E-03  31 |  | 2.06E-02  29 |  |  | 5.91E-07  31 |  |  |
| GO:0045780 | positive regulation of bone resorption |  |  | 1.88E-02  2 | 5.88E-04  2 |  |  |  |  |
| GO:0045787 | positive regulation of cell cycle |  |  | 2.40E-02  4 |  |  |  |  | 8.28E-03  3 |
| GO:0048522 | positive regulation of cellular process | 1.10E-03  30 |  | 1.46E-02  28 |  |  | 6.42E-07  29 |  |  |
| GO:0012501 | programmed cell death | 2.50E-03  21 |  | 2.06E-02  20 | 3.50E-05  23 |  |  |  |  |
| GO:0009150 | purine ribonucleotide metabolic process |  |  | 2.53E-02  6 | 8.99E-04  6 |  |  |  |  |
| GO:0042981 | regulation of apoptosis |  |  |  | 2.04E-04  18 |  | 2.76E-05  16 |  | 3.31E-02  3 |
| GO:0010941 | regulation of cell death |  |  |  | 2.39E-04  18 |  | 3.26E-05  16 |  |  |
| GO:0042127 | regulation of cell proliferation |  |  | 1.88E-02  16 | 3.04E-04  17 |  | 1.08E-05  17 |  |  |
| GO:0060334 | regulation of interferon-gamma-mediated signaling pathway |  |  | 1.46E-02  2 | 1.78E-04  2 |  |  |  | 1.09E-03  2 |
| GO:0043067 | regulation of programmed cell death |  |  |  | 2.27E-04  18 |  | 3.16E-05  16 |  |  |
| GO:0060338 | regulation of type I interferon-mediated signaling pathway |  |  | 1.16E-02  2 | 6.00E-05  2 |  | 3.72E-05  2 |  | 1.09E-03  2 |
| GO:0042221 | response to chemical stimulus | 4.00E-04  31 |  | 1.16E-02  24 | 1.00E-06  29 |  | 8.03E-09  29 |  |  |
| GO:0009725 | response to hormone stimulus | 2.40E-03  12 |  |  | 2.26E-04  11 |  | 2.05E-05  11 |  |  |
| GO:0010033 | response to organic substance | 3.00E-04  25 |  |  | 9.40E-05  12 |  | 2.59E-09  22 |  |  |
| GO:0051789 | response to protein stimulus |  |  | 2.53E-02  5 | 3.74E-04  6 |  |  |  |  |
| GO:0006986 | response to unfolded protein |  |  | 2.88E-02  4 |  |  |  |  | 3.88E-02  2 |
| GO:0060337 | type I interferon-mediated signaling pathway |  |  | 1.46E-02  2 | 1.78E-04  2 |  |  |  |  |
| Number of significant categories only with this tool | | 0 | 1 | 15 | 5 | 0 | 5 | 0 | 44 |

**Table S4B.** Results for Gene Ontology Molecular Function categories.

| **ID** | **Category** | **GOTM** | **WebGestalt** | **ToppFun** | **FatiGO** | **g:Profiler** | **DAVID** | **GeneCodis** |
| --- | --- | --- | --- | --- | --- | --- | --- | --- |
| **Total number of significant categories** | | **10** | **25** | **17** | **6** | **1** | **1** | **35** |
| GO:0030246 | carbohydrate binding |  | 1.73E-02  9 | 1.76E-03  9 | 2.70E-03  10 |  |  |  |
| GO:0019829 | cation-transporting ATPase activity | 3.14E-02  3 | 2.04E-02  3 | 1.76E-03  3 |  |  |  |  |
| GO:0051087 | chaperone binding | 2.38E-02  3 | 1.45E-02  3 | 6.01E-04  3 |  |  |  | 1.06E-03  3 |
| GO:0044212 | DNA regulatory region binding |  | 1.45E-02  4 | 6.86E-04  4 |  |  |  |  |
| GO:0005201 | extracellular matrix structural constituent |  | 3.60E-02  4 |  | 2.30E-02  5 |  |  | 1.18E-03  4 |
| GO:0005539 | glycosaminoglycan binding |  | 1.45E-02  6 | 8.04E-04  6 | 5.19E-03  7 |  |  |  |
| GO:0008201 | heparin binding |  | 1.49E-02  5 | 1.53E-03  5 | 2.97E-02  6 |  |  | 2.42E-03  4 |
| GO:0015078 | hydrogen ion transmembrane transporter activity | 9.20E-03  6 | 8.00E-03  6 | 8.50E-05  6 |  |  |  |  |
| GO:0046933 | hydrogen ion transporting ATP synthase activity, rotational mechanism |  | 4.06E-02  2 |  |  |  |  | 7.39E-03  2 |
| GO:0022890 | inorganic cation transmembrane transporter activity | 3.14E-02  6 | 1.45E-02  6 | 1.36E-03  6 |  |  |  |  |
| GO:0005159 | insulin-like growth factor receptor binding |  | 3.85E-02  2 |  |  |  |  | 6.70E-03  2 |
| GO:0015077 | monovalent inorganic cation transmembrane transporter activity | 1.11E-02  6 | 8.00E-03  6 | 1.77E-04  6 |  |  |  |  |
| GO:0050998 | nitric-oxide synthase binding | 2.78E-02  2 |  | 1.43E-03  2 |  |  |  | 3.97E-03  2 |
| GO:0001871 | pattern binding |  | 1.49E-02  6 | 3.37E-04  7 | 2.70E-03  7 |  |  |  |
| GO:0030247 | polysaccharide binding |  | 1.49E-02  6 | 1.28E-03  6 | 3.93E-03  7 |  |  |  |
| GO:0010843 | promoter binding | 6.20E-03  4 | 1.45E-02  4 | 8.40E-04  4 |  |  |  |  |
| GO:0005515 | protein binding | 6.20E-03  80 | 1.29E-02  80 |  |  | 6.13E-06  78 | 1.56E-02  69 | 5.14E-10  47 |
| GO:0046961 | proton-transporting ATPase activity, rotational mechanism | 1.23E-02  3 | 1.29E-02  3 | 3.93E-04  3 |  |  |  | 1.06E-03  3 |
| GO:0003723 | RNA binding |  | 3.32E-02  13 |  |  |  |  | 4.11E-03  8 |
| GO:0003697 | single-stranded DNA binding |  | 4.06E-02  3 | 7.35E-04  4 |  |  |  | 4.78E-03  3 |
| GO:0003735 | structural constituent of ribosome |  | 4.77E-02  5 |  |  |  |  | 2.33E-03  5 |
| GO:0070644 | vitamin D response element binding | 6.20E-03  2 | 1.07E-02  2 | 1.57E-04  2 |  |  |  |  |
| Number of significant categories only with this tool | | 0 | 4 | 1 | 0 | 0 | 0 | 24 |

**Table S4C. Results for KEGG pathway categories.**

| **ID** | **Category** | **GATHER** | **WebGestalt** | **ConsensusPathDB** | **ToppFun** | **g:Profiler** | **DAVID** | **GeneCodis** |
| --- | --- | --- | --- | --- | --- | --- | --- | --- |
| **Total number of significant categories** | | **0** | **34** | **2** | **1** | **1** | **0** | **21** |
| KEGG5010 | Alzheimer's disease |  | 5.41E-05  6 |  |  |  |  | 4.17E-04  6 |
| KEGG970 | Aminoacyl-tRNA biosynthesis |  | 1.15E-02  2 |  |  |  |  | 3.46E-02  2 |
| KEGG5014 | Amyotrophic lateral sclerosis (ALS) |  | 1.44E-02  2 |  |  |  |  | 4.35E-02  2 |
| KEGG5217 | Basal cell carcinoma |  | 1.44E-02  2 |  |  |  |  | 4.35E-02  2 |
| KEGG4512 | ECM-receptor interaction |  | 3.31E-05  5 | 2.98E-02  5 |  |  |  | 1.83E-03  4 |
| KEGG4510 | focal adhesion |  | 9.00E-04  5 |  |  |  |  | 1.91E-02  4 |
| KEGG5016 | Huntington's disease |  | 7.54E-05  6 |  |  |  |  | 4.46E-04  6 |
| KEGG5223 | Non-small cell lung cancer |  | 1.44E-02  2 |  |  |  |  | 4.65E-02  2 |
| KEGG190 | oxidative phosphorylation |  | 3.49E-06  7 | 2.38E-02  7 | 4.90E-05  6 | 3.87E-04  7 |  | 9.18E-06  7 |
| KEGG4115 | p53 signaling pathway |  | 2.70E-03  3 |  |  |  |  | 1.08E-02  3 |
| KEGG5012 | Parkinson's disease |  | 2.00E-04  5 |  |  |  |  | 7.61E-04  5 |
| KEGG5200 | pathways in cancer |  | 3.31E-05  8 |  |  |  |  | 6.03E-03  6 |
| KEGG4810 | Regulation of actin cytoskeleton |  | 2.73E-02  3 |  |  |  |  | 2.19E-02  4 |
| KEGG3010 | ribosome |  | 5.00E-04  4 |  |  |  |  | 1.66E-02  3 |
| KEGG5222 | Small cell lung cancer |  | 4.20E-03  3 |  |  |  |  | 1.78E-02  3 |
| KEGG4940 | Type I diabetes mellitus |  | 1.15E-02  2 |  |  |  |  | 3.40E-02  2 |
| KEGG5110 | Vibrio cholerae infection |  | 1.90E-03  3 |  |  |  |  | 8.13E-03  3 |
| Number of significanat categories only with this tool | | 0 | 17 | 0 | 0 | 0 | 0 | 4 |
