# Supplementary material for: Consensus Pathways Implicated in Prognosis of Colorectal Cancer Identified Through Systematic Enrichment Analysis of Gene Expression Profiling Studies
Source: PLoS One. 2011 Apr 25;6(4):e18867. doi: 10.1371/journal.pone.0018867 (PMC3081819; doi:10.1371/journal.pone.0018867)
Supplement: Table S5 — Results of all enrichment tools used with the 1475 gene list. Only those categories selected by at least two enrichment tools are shown. In each case, the first row represents the overrepresentation P value adjusted for multiple testing, and the second row the number of genes in the category within the 1475 gene list. Table S5A. Results for Gene Ontology Biological Process categories; Table S5B. Results for Gene Ontology Molecular Function categories; Table S5C. Results for KEGG pathway categories. (DOC) [file pone.0018867.s007.doc]

**Table S5. Results of all enrichment tools used with the 1475 gene list.**

Only those categories selected by at least two enrichment tools are shown. In each case, the first row represents the overrepresentation P value adjusted for multiple testing, and the second row the number of genes in the category within the 1475 gene list.

**Table S5A.** Results for Gene Ontology Biological Process categories.

| **ID** | **Category** | **GOTM** | **GATHER** | **WebGestalt** | **ToppFun** | **FatiGO** | **g:Profiler** | **DAVID** | **GeneCodis** |
| --- | --- | --- | --- | --- | --- | --- | --- | --- | --- |
| **Total number of significant categories** | | **10** | **11** | **40** | **234** | **53** | **181** | **95** | **115** |
| GO:0030036 | actin cytoskeleton organization |  |  |  | 3.01E-04  43 |  |  | 3.28E-02  32 | 5.95E-07  17 |
| GO:0030029 | actin filament-based process |  |  |  | 1.94E-04  46 |  |  | 4.31E-02  33 |  |
| GO:0031145 | anaphase-promoting complex-dependent proteasomal ubiquitin-dependent protein catabolic process |  |  | 5.28E-07  22 | 0.00E+00  22 |  | 2.58E-08  20 |  | 8.38E-14  20 |
| GO:0048856 | anatomical structure development |  |  |  |  | 1.50E-03  213 | 9.06E-17  289 | 2.82E-04  225 |  |
| GO:0010926 | anatomical structure formation |  |  |  | 0.00E+00  187 |  | 7.22E-11  152 |  |  |
| GO:0009653 | anatomical structure morphogenesis |  |  |  |  | 2.64E-02  111 | 1.01E-08  138 |  |  |
| GO:0006916 | anti-apoptosis |  |  |  |  |  |  | 3.92E-02  27 | 3.03E-09  25 |
| GO:0006915 | apoptosis |  |  |  | 0.00E+00  152 |  |  | 1.61E-04  101 | 1.17E-12  45 |
| GO:0007610 | behavior |  |  |  |  | 1.34E-02  44 | 1.57E-06  67 |  |  |
| GO:0022610 | biological adhesion |  |  |  | 8.00E-06  111 |  | 2.55E-06  96 | 3.40E-02  87 |  |
| GO:0065007 | biological regulation |  |  |  |  |  | 1.32E-12  733 | 8.53E-05  491 |  |
| GO:0009058 | biosynthetic process |  |  |  |  | 1.16E-02  146 | 3.85E-10  234 | 4.18E-02  154 |  |
| GO:0001568 | blood vessel development |  |  |  | 1.30E-05  51 |  | 2.97E-06  37 |  |  |
| GO:0060348 | bone development |  |  |  | 4.30E-05  35 |  | 5.66E-07  29 |  |  |
| GO:0007155 | cell adhesion |  |  |  | 7.00E-06  111 | 3.52E-03  87 | 2.42E-06  96 | 3.40E-02  87 | 2.96E-14  53 |
| GO:0007049 | cell cycle |  |  |  | 0.00E+00  139 | 1.34E-02  96 |  | 5.32E-03  103 | 2.23E-09  42 |
| GO:0022402 | cell cycle process |  |  |  | 4.00E-06  90 | 1.43E-02  87 | 1.66E-07  74 | 5.74E-04  94 |  |
| GO:0008219 | cell death |  |  |  | 0.00E+00  164 | 2.18E-02  96 |  | 3.07E-04  104 |  |
| GO:0030154 | cell differentiation |  |  |  |  |  | 7.23E-08  173 | 1.26E-02  185 | 7.55E-10  44 |
| GO:0016477 | cell migration |  |  |  | 3.30E-05  62 |  |  |  | 1.70E-04  9 |
| GO:0048870 | cell motility |  |  |  | 5.80E-05  66 | 5.29E-03  54 |  | 2.90E-04  62 |  |
| GO:0006928 | cell motion |  |  |  | 1.00E-06  93 |  | 3.11E-07  70 |  |  |
| GO:0008283 | cell proliferation | 6.16E-11  161 | 1.41E-05  150 | 1.47E-08  155 | 0.00E+00  175 | 9.22E-07  112 | 1.06E-06  56 | 5.83E-12  129 | 8.47E-14  37 |
| GO:0044249 | cellular biosynthetic process |  |  |  |  | 1.08E-02  137 | 1.04E-09  225 | 1.24E-03  131 |  |
| GO:0030003 | cellular cation homeostasis |  |  |  | 2.40E-05  46 |  | 5.88E-07  42 |  |  |
| GO:0022607 | cellular component assembly |  |  |  | 2.00E-06  142 |  | 1.43E-08  121 | 3.27E-02  71 |  |
| GO:0044085 | cellular component biogenesis |  |  |  | 0.00E+00  160 |  | 5.91E-10  136 |  |  |
| GO:0006928 | cellular component movement |  |  | 2.01E-06  88 |  |  |  |  | 8.09E-12  22 |
| GO:0016043 | cellular component organization |  |  |  |  | 1.82E-03  260 | 1.11E-14  285 | 8.81E-05  280 |  |
| GO:0048869 | cellular developmental process |  |  |  |  | 2.89E-02  211 | 1.63E-08  180 | 1.26E-02  185 |  |
| GO:0051641 | cellular localization |  |  |  | 1.80E-05  145 | 2.46E-03  105 | 7.38E-07  119 | 3.13E-04  111 |  |
| GO:0044260 | cellular macromolecule metabolic process |  |  |  |  | 4.47E-05  336 | 7.19E-19  430 | 9.50E-06  357 |  |
| GO:0044237 | cellular metabolic process |  |  |  |  | 1.32E-02  691 | 1.59E-27  589 | 5.36E-03  711 |  |
| GO:0009987 | cellular process |  |  |  |  |  | 5.27E-38  1044 | 2.73E-04  1069 |  |
| GO:0043623 | cellular protein complex assembly |  |  |  | 1.00E-06  43 |  | 4.32E-07  33 |  |  |
| GO:0044267 | cellular protein metabolic process | 6.16E-11  339 |  | 5.41E-08  303 |  | 1.99E-04  333 | 6.40E-16  293 | 6.35E-06  354 |  |
| GO:0070887 | cellular response to chemical stimulus |  |  |  | 1.10E-05  24 |  | 4.19E-10  45 |  |  |
| GO:0032870 | cellular response to hormone stimulus |  |  |  | 9.60E-05  27 |  | 5.90E-08  27 |  |  |
| GO:0051716 | cellular response to stimulus |  |  |  | 2.60E-05  100 |  | 2.63E-08  95 |  |  |
| GO:0006935 | chemotaxis |  |  |  |  |  | 8.36E-07  35 | 3.34E-02  25 | 1.23E-10  23 |
| GO:0016265 | death |  |  |  | 0.00E+00  164 | 1.50E-03  96 |  | 3.07E-04  104 |  |
| GO:0046697 | decidualization |  |  |  | 6.10E-05  7 |  |  |  | 8.55E-07  7 |
| GO:0032502 | developmental process | 1.48E-11  414 |  |  |  |  | 5.62E-18  349 | 2.24E-06  338 |  |
| GO:0007167 | enzyme linked receptor protein signaling pathway |  |  |  | 0.00E+00  76 | 1.09E-02  43 | 1.57E-08  54 | 2.05E-05  51 |  |
| GO:0051649 | establishment of cellular localization |  |  |  |  | 3.85E-03  104 |  | 2.77E-04  109 |  |
| GO:0051234 | establishment of localization |  |  |  |  |  | 1.22E-07  289 | 2.39E-02  264 |  |
| GO:0051649 | establishment of localization in cell |  |  |  | 7.00E-06  140 |  | 8.91E-07  112 |  |  |
| GO:0045184 | establishment of protein localization |  |  |  | 2.50E-05  111 | 1.31E-02  83 |  | 2.03E-03  91 |  |
| GO:0006091 | generation of precursor metabolites and energy |  |  |  | 0.00E+00  62 | 1.59E-02  95 | 2.18E-09  60 | 4.50E-03  80 |  |
| GO:0048534 | hemopoietic or lymphoid organ development |  |  |  | 3.00E-06  63 |  | 3.88E-06  41 | 3.99E-02  31 |  |
| GO:0042592 | homeostatic process |  |  |  | 4.00E-06  116 |  | 7.00E-07  110 |  |  |
| GO:0006818 | hydrogen transport |  |  |  | 5.40E-04  15 | 3.53E-02  16 |  |  |  |
| GO:0006955 | immune response |  |  |  | 0.00E+00  111 | 1.50E-03  93 |  |  | 6.46E-25  65 |
| GO:0002520 | immune system development |  |  |  | 0.00E+00  68 |  | 2.16E-06  43 | 2.88E-02  33 |  |
| GO:0002376 | immune system process |  |  | 3.33E-08  154 | 0.00E+00  167 |  |  |  |  |
| GO:0006917 | induction of apoptosis |  |  |  | 3.10E-04  50 |  |  |  | 6.07E-08  22 |
| GO:0006954 | inflammatory response |  |  |  | 5.60E-05  57 |  |  |  | 3.07E-09  28 |
| GO:0044419 | interspecies interaction between organisms |  |  |  | 2.00E-06  51 |  |  |  | 2.77E-20  48 |
| GO:0006886 | intracellular protein transport |  |  |  | 0.00E+00  74 | 4.46E-03  61 |  | 3.62E-05  64 | 2.81E-11  28 |
| GO:0007242 | intracellular signaling cascade |  |  |  |  |  | 6.40E-08  153 |  | 7.58E-07  27 |
| GO:0046907 | intracellular transport |  |  | 4.37E-07  106 | 0.00E+00  120 | 9.45E-05  94 | 9.10E-07  96 | 9.45E-05  95 |  |
| GO:0051179 | localization |  |  |  |  |  | 5.34E-11  349 | 1.76E-04  315 |  |
| GO:0051674 | localization of cell |  |  |  | 1.00E-06  93 | 1.82E-03  54 | 3.11E-07  70 | 2.90E-04  62 |  |
| GO:0040011 | locomotion |  |  |  | 2.00E-06  85 |  | 2.00E-08  72 |  |  |
| GO:0065003 | macromolecular complex assembly |  |  |  | 4.00E-06  113 |  | 5.37E-07  95 | 4.64E-02  66 |  |
| GO:0043933 | macromolecular complex subunit organization |  |  |  | 8.00E-06  119 |  | 8.71E-07  99 |  |  |
| GO:0033036 | macromolecule localization |  |  |  | 3.30E-05  140 |  |  | 8.39E-04  102 |  |
| GO:0043170 | macromolecule metabolic process |  |  |  |  |  | 3.56E-18  478 | 1.52E-02  624 |  |
| GO:0008152 | metabolic process |  |  |  |  |  | 4.80E-25  672 | 1.75E-03  786 |  |
| GO:0000278 | mitotic cell cycle |  |  |  | 1.00E-06  75 |  | 6.15E-09  57 |  |  |
| GO:0007275 | multicellular organismal development |  |  |  |  | 2.89E-02  214 | 1.92E-14  313 | 5.93E-03  232 | 1.77E-14  75 |
| GO:0051704 | multi-organism process |  |  |  | 0.00E+00  108 |  | 1.88E-08  94 |  |  |
| GO:0043066 | negative regulation of apoptosis |  |  |  | 4.80E-05  59 |  |  | 3.29E-02  34 | 4.30E-06  16 |
| GO:0048519 | negative regulation of biological process |  |  |  |  | 2.05E-04  141 | 2.35E-13  206 | 1.53E-05  145 |  |
| GO:0043086 | negative regulation of catalytic activity |  |  |  | 0.00E+00  52 |  | 1.79E-10  50 |  |  |
| GO:0045596 | negative regulation of cell differentiation |  |  |  | 1.20E-05  40 |  | 9.99E-08  38 |  |  |
| GO:0008285 | negative regulation of cell proliferation |  |  |  | 6.00E-04  52 |  |  | 3.67E-02  35 | 6.71E-13  36 |
| GO:0031324 | negative regulation of cellular metabolic process |  |  |  | 4.11E-04  92 |  | 3.08E-07  86 |  |  |
| GO:0048523 | negative regulation of cellular process |  |  |  |  | 1.61E-03  129 | 4.75E-12  187 | 2.38E-05  139 |  |
| GO:0032269 | negative regulation of cellular protein metabolic process |  |  |  | 2.50E-05  33 |  | 6.58E-07  31 |  |  |
| GO:0051093 | negative regulation of developmental process |  |  |  | 1.00E-06  92 |  | 3.09E-09  82 |  |  |
| GO:0051352 | negative regulation of ligase activity |  |  | 8.83E-07  22 | 0.00E+00  22 |  | 4.41E-08  20 |  |  |
| GO:0044092 | negative regulation of molecular function |  |  | 1.41E-06  59 |  |  | 2.23E-11  58 |  |  |
| GO:0043069 | negative regulation of programmed cell death |  |  |  | 3.60E-05  60 |  |  | 3.75E-02  34 |  |
| GO:0051248 | negative regulation of protein metabolic process |  |  |  | 2.80E-05  34 |  | 7.87E-07  32 |  |  |
| GO:0051444 | negative regulation of ubiquitin-protein ligase activity |  |  | 8.83E-07  22 | 0.00E+00  0 |  | 4.41E-08  20 |  |  |
| GO:0051436 | negative regulation of ubiquitin-protein ligase activity during mitotic cell cycle |  |  | 1.77E-06  21 | 0.00E+00  21 |  | 1.42E-07  19 |  | 7.34E-13  19 |
| GO:0048513 | organ development |  |  |  |  | 3.85E-03  142 | 6.67E-16  218 | 1.59E-04  149 |  |
| GO:0009887 | organ morphogenesis |  |  |  | 1.00E-05  131 |  | 3.75E-06  85 |  | 1.24E-06  17 |
| GO:0006996 | organelle organization |  |  |  |  |  | 3.23E-08  156 | 1.58E-03  134 |  |
| GO:0001503 | ossification |  |  |  | 6.70E-05  33 |  | 5.09E-07  134 |  | 1.73E-05  10 |
| GO:0006119 | oxidative phosphorylation |  |  |  | 1.00E-06  25 |  | 1.33E-06  26 | 3.45E-02  21 | 1.81E-04  4 |
| GO:0018193 | peptidyl-amino acid modification |  |  |  | 0.00E+00  49 |  | 1.83E-06  28 |  |  |
| GO:0018108 | peptidyl-tyrosine phosphorylation |  |  |  | 1.20E-05  25 |  |  |  | 2.45E-08  11 |
| GO:0006796 | phosphate metabolic process | 1.23E-10  187 |  | 1.11E-07  168 | 0.00E+00  156 | 1.61E-03  117 | 6.18E-13  145 | 1.87E-04  125 |  |
| GO:0006793 | phosphorus metabolic process |  |  | 1.11E-07  168 | 0.00E+00  156 | 4.80E-04  117 | 6.18E-13  145 | 1.87E-04  125 |  |
| GO:0016310 | phosphorylation |  |  | 1.42E-07  149 | 0.00E+00  136 | 9.46E-03  97 | 5.31E-12  123 | 2.92E-04  107 |  |
| GO:0043065 | positive regulation of apoptosis |  |  |  | 3.00E-05  66 |  | 1.28E-06  54 |  |  |
| GO:0048518 | positive regulation of biological process | 2.67E-15  258 |  | 9.74E-11  251 |  | 2.17E-06  153 | 4.74E-24  255 | 1.11E-10  158 |  |
| GO:0009891 | positive regulation of biosynthetic process |  |  |  | 5.30E-05  95 |  | 7.00E-09  92 |  |  |
| GO:0045780 | positive regulation of bone resorption |  |  |  | 2.38E-04  4 |  |  |  | 1.75E-05  4 |
| GO:0043085 | positive regulation of catalytic activity |  |  | 1.47E-08  88 | 0.00E+00  91 |  | 7.19E-13  83 | 3.32E-02  35 |  |
| GO:0010942 | positive regulation of cell death |  |  |  | 4.20E-05  66 |  | 1.89E-06  54 |  |  |
| GO:0045597 | positive regulation of cell differentiation |  |  |  | 2.00E-06  44 |  | 1.37E-09  44 |  |  |
| GO:0030335 | positive regulation of cell migration |  |  |  | 2.84E-04  19 |  |  |  | 2.33E-09  15 |
| GO:0051272 | positive regulation of cell motion |  |  |  | 4.70E-05  22 |  | 1.74E-06  21 |  |  |
| GO:0008284 | positive regulation of cell proliferation |  | 8.44E-05  27 | 1.68E-06  68 | 0.00E+00  71 | 7.29E-03  39 | 4.81E-10  67 | 9.60E-07  49 | 1.98E-17  41 |
| GO:0031328 | positive regulation of cellular biosynthetic process |  |  |  | 3.50E-05  95 |  | 4.00E-09  92 |  |  |
| GO:0031325 | positive regulation of cellular metabolic process |  |  |  | 1.00E-06  118 |  | 1.55E-11  113 | 4.85E-02  52 |  |
| GO:0048522 | positive regulation of cellular process | 2.26E-16  243 |  | 2.99E-11  236 |  | 1.99E-04  121 | 3.92E-25  239 | 4.82E-10  144 |  |
| GO:0051094 | positive regulation of developmental process |  |  |  | 0.00E+00  109 |  | 1.15E-08  50 |  |  |
| GO:0033674 | positive regulation of kinase activity |  |  |  | 1.00E-06  45 |  | 1.16E-07  39 | 5.30E-03  26 |  |
| GO:0051351 | positive regulation of ligase activity |  |  |  | 0.00E+00  21 |  | 9.14E-07  19 |  |  |
| GO:0010604 | positive regulation of macromolecule metabolic process |  |  |  | 1.71E-04  105 |  | 1.11E-08  102 |  |  |
| GO:0043406 | positive regulation of MAP kinase activity |  |  |  | 1.98E-04  22 |  |  | 1.51E-02  16 |  |
| GO:0009893 | positive regulation of metabolic process |  |  |  | 4.00E-06  120 |  | 2.70E-11  116 | 1.16E-02  58 |  |
| GO:0044093 | positive regulation of molecular function |  |  | 1.11E-07  92 | 1.08E-04  48 |  | 1.65E-12  89 |  |  |
| GO:0045639 | positive regulation of myeloid cell differentiation |  |  |  | 4.00E-06  15 |  | 3.22E-07  12 |  |  |
| GO:0045935 | positive regulation of nucleobase, nucleoside, nucleotide and nucleic acid metabolic process |  |  |  | 2.27E-04  83 |  | 3.09E-08  82 | 4.98E-02  44 |  |
| GO:0043068 | positive regulation of programmed cell death |  |  |  | 3.70E-05  66 |  | 1.62E-06  54 |  |  |
| GO:0045860 | positive regulation of protein kinase activity |  |  |  | 1.00E-06  44 |  | 4.85E-08  39 | 8.45E-03  25 |  |
| GO:0045941 | positive regulation of transcription |  |  |  |  |  | 5.35E-07  72 | 3.32E-02  44 | 2.66E-09  21 |
| GO:0045944 | positive regulation of transcription from RNA polymerase II promoter |  |  |  |  |  | 4.27E-06  51 |  | 7.77E-14  39 |
| GO:0051347 | positive regulation of transferase activity |  |  |  | 1.00E-06  46 |  | 1.04E-07  40 | 3.03E-03  40 |  |
| GO:0051443 | positive regulation of ubiquitin-protein ligase activity |  |  |  | 0.00E+00  21 |  | 4.71E-07  19 |  |  |
| GO:0051437 | positive regulation of ubiquitin-protein ligase activity during mitotic cell cycle |  |  |  | 0.00E+00  21 |  | 2.96E-07  19 |  | 1.69E-12  19 |
| GO:0043687 | post-translational protein modification |  |  |  | 2.00E-06  171 |  | 6.21E-09  146 |  |  |
| GO:0044238 | primary metabolic process |  |  |  |  |  | 2.97E-21  591 | 7.81E-03  711 |  |
| GO:0012501 | programmed cell death |  |  |  | 0.00E+00  154 |  |  | 1.93E-04  101 |  |
| GO:0010498 | proteasomal protein catabolic process |  |  | 1.49E-06  29 | 0.00E+00  30 |  | 2.10E-08  25 |  |  |
| GO:0043161 | proteasomal ubiquitin-dependent protein catabolic process |  |  | 1.49E-06  29 | 0.00E+00  30 |  | 2.10E-08  25 |  |  |
| GO:0046777 | protein amino acid autophosphorylation |  |  |  | 3.77E-04  19 |  |  |  | 4.27E-05  12 |
| GO:0006468 | protein amino acid phosphorylation |  |  |  | 2.00E-06  105 |  | 7.52E-08  94 | 3.00E-02  81 | 4.21E-08  38 |
| GO:0006461 | protein complex assembly |  |  |  | 1.00E-06  79 |  | 1.18E-07  68 | 4.71E-02  40 | 2.62E-08  18 |
| GO:0070271 | protein complex biogenesis |  |  |  | 1.00E-06  79 |  | 1.18E-07  68 |  |  |
| GO:0006457 | protein folding |  |  |  | 3.78E-04  29 |  |  |  | 8.44E-09  22 |
| GO:0007243 | protein kinase cascade |  |  |  | 1.40E-05  79 |  |  | 2.97E-02  51 |  |
| GO:0008104 | protein localization |  |  |  | 5.40E-05  123 | 1.15E-02  85 |  | 2.38E-03  95 |  |
| GO:0019538 | protein metabolic process |  |  | 1.05E-07  345 |  | 3.65E-05  349 | 3.56E-15  339 | 5.41E-07  379 |  |
| GO:0006605 | protein targeting |  |  |  | 1.00E-06  48 |  |  | 1.98E-04  40 |  |
| GO:0015031 | protein transport |  |  |  | 8.80E-05  107 | 3.53E-02  78 |  | 2.39E-03  86 | 3.66E-05  30 |
| GO:0015992 | proton transport |  |  |  | 3.87E-04  15 |  |  |  | 2.87E-09  15 |
| GO:0042981 | regulation of apoptosis |  |  | 1.21E-06  117 | 0.00E+00  129 |  | 2.03E-12  110 | 2.29E-04  75 | 1.65E-08  19 |
| GO:0050789 | regulation of biological process |  |  |  |  | 1.32E-02  420 | 6.41E-09  672 | 1.54E-04  449 |  |
| GO:0065008 | regulation of biological quality |  |  |  |  |  | 1.92E-11  195 | 1.60E-02  99 |  |
| GO:0050790 | regulation of catalytic activity |  |  | 1.47E-08  127 | 0.00E+00  133 |  | 7.94E-15  123 |  |  |
| GO:0030155 | regulation of cell adhesion |  |  |  | 5.00E-06  33 |  | 3.61E-06  26 |  | 4.36E-06  8 |
| GO:0051726 | regulation of cell cycle |  |  |  | 3.00E-06  67 | 7.48E-03  72 | 1.75E-07  49 | 5.80E-04  72 | 3.49E-08  12 |
| GO:0010941 | regulation of cell death |  |  | 8.83E-07  119 | 0.00E+00  131 |  | 2.17E-12  111 |  |  |
| GO:0060284 | regulation of cell development |  |  |  | 1.40E-04  35 |  | 4.60E-07  35 |  |  |
| GO:0045595 | regulation of cell differentiation |  |  | 1.26E-07  80 | 0.00E+00  90 |  | 2.22E-14  86 |  |  |
| GO:0030334 | regulation of cell migration |  |  |  | 4.40E-05  32 |  | 2.51E-07  31 |  |  |
| GO:0051270 | regulation of cell motion |  |  |  | 1.80E-05  36 |  | 1.81E-07  34 |  |  |
| GO:0042127 | regulation of cell proliferation |  | 1.41E-05  48 | 1.11E-07  114 | 0.00E+00  121 | 5.37E-04  69 | 1.36E-13  116 | 3.71E-08  84 |  |
| GO:0051128 | regulation of cellular component organization |  |  |  | 0.00E+00  110 |  | 6.05E-10  70 |  |  |
| GO:0050794 | regulation of cellular process |  | 1.41E-05  114 |  |  |  | 1.19E-08  650 | 2.51E-03  409 |  |
| GO:0032268 | regulation of cellular protein metabolic process |  |  |  | 0.00E+00  79 |  | 5.41E-09  73 |  |  |
| GO:0050793 | regulation of developmental process | 9.16E-15  197 |  | 1.11E-07  102 |  | 1.49E-02  35 | 3.08E-16  140 |  |  |
| GO:0051052 | regulation of DNA metabolic process |  |  |  | 1.12E-04  24 |  | 1.16E-06  25 |  |  |
| GO:0002682 | regulation of immune system process |  |  |  | 4.00E-06  66 |  | 2.96E-06  57 |  |  |
| GO:0043549 | regulation of kinase activity |  |  |  | 0.00E+00  65 |  | 1.57E-09  57 | 7.60E-04  40 |  |
| GO:0051340 | regulation of ligase activity |  |  | 1.64E-06  24 | 0.00E+00  24 |  | 5.11E-08  22 |  |  |
| GO:0032879 | regulation of localization |  |  |  | 0.00E+00  94 |  | 1.35E-10  88 |  |  |
| GO:0040012 | regulation of locomotion |  |  |  | 8.00E-06  37 |  | 7.52E-08  35 |  |  |
| GO:0043405 | regulation of MAP kinase activity |  |  |  | 1.40E-05  30 |  |  | 5.35E-03  21 |  |
| GO:0065009 | regulation of molecular function |  |  | 3.69E-08  139 | 0.00E+00  149 |  | 1.57E-15  137 |  |  |
| GO:0051239 | regulation of multicellular organismal process |  |  |  | 2.00E-06  134 |  | 3.67E-12  127 |  |  |
| GO:0051960 | regulation of nervous system development |  |  |  | 1.83E-04  33 |  | 1.49E-06  33 |  |  |
| GO:0050767 | regulation of neurogenesis |  |  |  | 2.02E-04  30 |  | 1.78E-06  30 |  |  |
| GO:0019220 | regulation of phosphate metabolic process |  |  | 9.46E-07  77 | 1.01E-04  33 |  | 1.49E-11  77 |  |  |
| GO:0051174 | regulation of phosphorus metabolic process |  |  | 9.46E-07  77 | 1.01E-04  33 |  | 1.49E-11  77 |  |  |
| GO:0042325 | regulation of phosphorylation |  |  | 1.43E-06  74 | 1.04E-04  32 |  | 5.19E-11  74 |  |  |
| GO:0043067 | regulation of programmed cell death |  |  | 1.09E-06  118 | 0.00E+00  130 |  | 1.85E-12  111 | 3.12E-04  75 |  |
| GO:0000074 | regulation of progression through cell cycle |  |  |  |  | 2.28E-02  71 |  | 5.23E-04  72 |  |
| GO:0045859 | regulation of protein kinase activity |  |  |  | 0.00E+00  64 |  | 4.58E-10  57 | 9.35E-04  39 |  |
| GO:0051246 | regulation of protein metabolic process |  |  |  | 0.00E+00  87 |  | 2.90E-09  81 |  |  |
| GO:0006357 | regulation of transcription from RNA polymerase II promoter |  |  |  |  |  | 1.05E-06  86 |  | 1.67E-04  17 |
| GO:0051338 | regulation of transferase activity |  |  | 4.70E-02 | 0.00E+00  67 |  | 8.72E-10  59 | 5.73E-04  41 |  |
| GO:0051438 | regulation of ubiquitin-protein ligase activity |  |  | 9.18E-07  24 | 0.00E+00  24 |  | 2.51E-08  22 |  |  |
| GO:0051439 | regulation of ubiquitin-protein ligase activity during mitotic cell cycle |  |  | 6.57E-07  23 | 0.00E+00  23 |  | 2.29E-08  21 |  |  |
| GO:0043200 | response to amino acid stimulus |  |  |  | 4.21E-04  7 |  |  |  | 4.89E-05  6 |
| GO:0042221 | response to chemical stimulus | 3.18E-19  250 |  | 3.62E-12  190 | 0.00E+00  214 | 1.50E-03  66 | 6.27E-27  212 | 5.60E-04  80 |  |
| GO:0042493 | response to drug |  |  |  | 4.28E-04  34 |  |  |  | 4.30E-11  29 |
| GO:0009719 | response to endogenous stimulus | 3.85E-11  80 |  | 1.97E-06  68 | 0.00E+00  73 |  | 2.83E-16  79 |  |  |
| GO:0043627 | response to estrogen stimulus |  |  |  | 4.53E-04  20 |  | 9.57E-07  23 |  | 1.78E-06  11 |
| GO:0009605 | response to external stimulus |  |  |  | 2.00E-06  130 | 4.55E-02  66 | 3.13E-08  133 |  |  |
| GO:0009725 | response to hormone stimulus |  |  |  | 0.00E+00  66 |  | 8.84E-15  71 |  |  |
| GO:0014070 | response to organic cyclic substance |  |  |  | 2.06E-04  23 |  |  |  | 1.86E-10  18 |
| GO:0010243 | response to organic nitrogen |  |  |  | 2.27E-04  15 |  |  |  | 1.63E-04  7 |
| GO:0010033 | response to organic substance | 6.60E-13  167 |  | 7.04E-08  109 | 0.00E+00  66 |  | 9.58E-19  126 |  |  |
| GO:0048545 | response to steroid hormone stimulus |  |  |  | 1.01E-04  33 |  | 2.89E-07  36 |  |  |
| GO:0006950 | response to stress |  |  | 5.28E-07  214 |  | 7.04E-04  123 | 8.12E-12  237 | 1.64E-05  135 |  |
| GO:0033273 | response to vitamin |  |  |  | 1.00E-06  21 |  | 2.33E-07  19 |  |  |
| GO:0042274 | ribosomal small subunit biogenesis |  |  |  | 2.14E-04  6 |  |  |  | 3.21E-06  6 |
| GO:0007165 | signal transduction |  |  |  |  |  | 7.68E-08  367 |  | 2.81E-31  149 |
| GO:0001501 | skeletal system development |  |  |  | 9.10E-05  57 |  | 1.19E-06  50 |  | 1.21E-05  15 |
| GO:0006930 | substrate-bound cell migration, cell extension |  |  |  | 2.38E-04  4 |  |  |  | 1.75E-05  4 |
| GO:0048731 | system development |  |  |  |  | 1.31E-02  175 | 9.98E-16  271 | 5.85E-04  188 |  |
| GO:0042330 | taxis |  |  |  |  | 3.15E-02  26 | 8.36E-07  35 | 3.34E-02  25 |  |
| GO:0009888 | tissue development |  |  |  | 1.00E-06  124 |  | 9.41E-07  86 |  |  |
| GO:0006366 | transcription from RNA polymerase II promoter |  |  |  | 2.87E-04  111 |  |  | 4.94E-02  76 | 4.91E-05  15 |
| GO:0006412 | translation |  |  |  | 0.00E+00  68 |  | 4.53E-06  65 |  | 1.21E-04  15 |
| GO:0006414 | translational elongation |  |  | 6.28E-08  32 | 0.00E+00  32 |  | 2.56E-10  30 |  | 9.02E-20  29 |
| GO:0007169 | transmembrane receptor protein tyrosine kinase signaling pathway |  |  |  | 5.30E-05  48 |  |  | 7.89E-04  35 |  |
| GO:0006810 | transport |  |  |  |  |  | 9.57E-08  288 |  | 4.77E-18  51 |
| GO:0006511 | ubiquitin-dependent protein catabolic process |  |  |  | 3.36E-04  38 |  |  |  | 2.71E-04  15 |
| GO:0001944 | vasculature development |  |  |  | 1.00E-05  52 |  | 2.10E-06  38 |  |  |
| Number of significant categories only with this tool | | 0 | 7 | 0 | 73 | 2 | 21 | 2 | 59 |

**Table S5B.** Results for Gene Ontology Molecular Function categories.

| **ID** | **Category** | **GOTM** | **WebGestalt** | **ToppFun** | **FatiGO** | **g:Profiler** | **DAVID** | **GeneCodis** |
| --- | --- | --- | --- | --- | --- | --- | --- | --- |
| **Total number of significant categories** | | **10** | **40** | **34** | **4** | **18** | **13** | **80** |
| GO:0005488 | binding | 5.89E-06  1130 | 2.65E-06  1106 |  |  | 6.88E-18  1114 | 4.79E-13  1071 | 3.29E-05  35 |
| GO:0019829 | cation-transporting ATPase activity |  | 5.30E-03  10 | 1.95E-04  10 |  |  |  |  |
| GO:0008009 | chemokine activity |  | 1.00E-03  14 | 1.30E-05  14 |  |  | 1.85E-02  14 | 1.37E-09  14 |
| GO:0042379 | chemokine receptor binding |  | 1.30E-03  14 | 2.90E-05  14 |  |  | 1.89E-02  14 |  |
| GO:0004129 | cytochrome-c oxidase activity |  | 1.30E-03  10 | 4.00E-05  10 |  | 4.05E-06  11 |  | 3.62E-08  10 |
| GO:0019899 | enzyme binding |  |  | 1.51E-04  70 |  | 1.59E-06  65 |  |  |
| GO:0050840 | extracellular matrix binding |  | 1.03E-02  8 |  |  |  |  | 2.35E-04  5 |
| GO:0005539 | glycosaminoglycan binding |  | 9.40E-03  24 |  |  |  |  | 1.03E-04  5 |
| GO:0015002 | heme-copper terminal oxidase activity | 3.00E-04  11 | 1.30E-03  10 | 4.00E-05  10 |  | 4.05E-06  11 |  |  |
| GO:0015078 | hydrogen ion transmembrane transporter activity | 6.78E-06  28 | 4.92E-06  25 | 0.00E+00  27 | 1.34E-03  27 | 9.84E-09  28 | 2.22E-03  29 |  |
| GO:0042802 | identical protein binding |  |  | 1.57E-04  81 |  |  |  | 2.42E-19  47 |
| GO:0022890 | inorganic cation transmembrane transporter activity | 1.19E-05  36 | 3.91E-06  34 | 0.00E+00  36 |  | 2.66E-08  36 | 2.29E-03  35 |  |
| GO:0005178 | integrin binding |  | 1.44E-02  13 | 1.18E-04  15 |  |  |  | 1.59E-06  12 |
| GO:0019900 | kinase binding | 8.42E-05  33 | 7.30E-03  28 | 5.20E-05  32 |  | 2.55E-07  33 |  |  |
| GO:0015077 | monovalent inorganic cation transmembrane transporter activity | 1.07E-05  29 | 5.83E-06  27 | 0.00E+00  28 | 1.51E-03  28 | 2.29E-08  29 | 2.12E-03  30 |  |
| GO:0016491 | oxidoreductase activity |  | 1.67E-02  79 |  |  |  |  | 6.68E-08  37 |
| GO:0016675 | oxidoreductase activity, acting on heme group of donors |  | 1.30E-03  10 | 4.00E-05  10 |  | 4.05E-06  11 |  |  |
| GO:0016676 | oxidoreductase activity, acting on heme group of donors, oxygen as acceptor |  | 1.30E-03  10 | 4.00E-05  10 |  | 4.05E-06  11 |  |  |
| GO:0046934 | phosphatidylinositol-4,5-bisphosphate 3-kinase activity |  | 5.30E-03  4 | 2.02E-04  4 |  |  |  | 5.01E-05  4 |
| GO:0015405 | P-P-bond-hydrolysis-driven transmembrane transporter activity |  | 5.30E-03  22 | 1.43E-04  23 |  |  |  |  |
| GO:0015399 | primary active transmembrane transporter activity |  | 5.30E-03  22 | 1.43E-04  23 |  |  |  |  |
| GO:0005515 | protein binding | 2.40E-24  843 | 9.22E-20  831 |  | 3.55E-09  602 | 4.13E-34  826 | 5.57E-33  743 | 5.33E-146  523 |
| GO:0032403 | protein complex binding | 5.26E-08  43 | 3.91E-06  41 | 0.00E+00  43 |  | 1.78E-11  43 |  | 2.07E-09  16 |
| GO:0019901 | protein kinase binding |  | 1.17E-02  24 | 1.86E-04  27 |  | 1.39E-06  28 |  | 3.22E-09  19 |
| GO:0004713 | protein tyrosine kinase activity |  | 7.30E-03  26 |  |  |  | 4.25E-02  32 | 1.54E-04  8 |
| GO:0046961 | proton-transporting ATPase activity, rotational mechanism |  | 1.03E-02  7 |  |  |  |  | 9.90E-06  7 |
| GO:0005102 | receptor binding |  | 9.00E-04  106 | 4.00E-06  115 |  |  |  | 8.45E-05  15 |
| GO:0005057 | receptor signaling protein activity |  | 1.60E-03  29 | 3.10E-05  31 |  |  |  |  |
| GO:0004716 | receptor signaling protein tyrosine kinase activity |  | 3.10E-03  7 | 8.20E-05  7 |  |  |  | 1.03E-04  5 |
| GO:0003723 | RNA binding | 2.92E-05  97 | 3.00E-04  94 | 4.00E-06  96 |  | 9.58E-08  94 | 1.45E-03  92 | 7.54E-24  71 |
| GO:0005048 | signal sequence binding |  | 1.03E-02  7 | 2.09E-04  8 |  |  |  |  |
| GO:0003697 | single-stranded DNA binding |  |  | 2.70E-05  15 |  |  |  | 1.48E-06  11 |
| GO:0003735 | structural constituent of ribosome |  | 1.59E-05  34 | 0.00E+00  35 |  |  |  | 1.58E-18  34 |
| GO:0005198 | structural molecule activity |  | 2.69E-06  91 | 0.00E+00  95 |  | 4.00E-07  113 | 1.41E-02  99 | 1.25E-05  20 |
| GO:0043566 | structure-specific DNA binding |  |  | 6.00E-06  29 |  |  | 4.02E-02  20 |  |
| GO:0004298 | threonine-type endopeptidase activity |  | 3.10E-03  8 | 9.50E-05  8 |  |  |  | 1.82E-04  6 |
| GO:0070003 | threonine-type peptidase activity |  | 3.10E-03  8 | 9.50E-05  8 |  |  |  |  |
| GO:0016563 | transcription activator activity |  |  |  |  |  | 1.73E-02  45 | 2.84E-08  24 |
| GO:0034713 | type I transforming growth factor beta receptor binding |  | 2.50E-03  5 | 6.40E-05  5 |  |  |  | 7.12E-06  5 |
| GO:0051082 | unfolded protein binding | 7.07E-06  30 | 1.00E-04  26 | 1.00E-06  26 | 1.42E-02  26 | 1.71E-07  28 | 5.41E-03  26 | 5.05E-15  26 |
| Number of significant categories only with this tool | | 0 | 5 | 2 | 0 | 2 | 0 | 56 |

**Table S5C.** Results for KEGG pathway categories.

| **ID** | **Category** | **GATHER** | **WebGestalt** | **ConsensusPathDB** | **ToppFun** | **g:Profiler** | **DAVID** | **GeneCodis** |
| --- | --- | --- | --- | --- | --- | --- | --- | --- |
| **Total number of significant categories** | | **1** | **136** | **1** | **10** | **4** | **5** | **116** |
| KEGG2010 | ABC transporters |  | 3.90E-03  6 |  |  |  |  | 2.48E-03  6 |
| KEGG5221 | acute myeloid leukemia |  | 1.71E-08  14 |  |  |  |  | 1.84E-09  14 |
| KEGG4520 | adherens junction |  | 2.06E-06  13 |  |  |  |  | 4.69E-07  13 |
| KEGG4920 | adipocytokine signaling pathway |  | 1.65E-05  11 |  |  |  |  | 6.62E-06  11 |
| KEGG5010 | Alzheimer's disease |  | 1.15E-17  35 |  |  |  |  | 6.85E-19  34 |
| KEGG970 | Aminoacyl-tRNA biosynthesis |  | 2.80E-03  6 |  |  |  |  | 1.77E-03  6 |
| KEGG5014 | amyotrophic lateral sclerosis (ALS) |  | 1.21E-05  10 |  |  |  |  | 3.71E-05  9 |
| KEGG4612 | antigen processing and presentation |  | 2.71E-11  20 |  |  |  |  | 3.07E-08  15 |
| KEGG4210 | apoptosis |  | 2.25E-11  20 |  |  |  |  | 2.47E-11  19 |
| KEGG590 | Arachidonic acid metabolism |  | 3.70E-03  7 |  |  |  |  | 1.97E-03  7 |
| KEGG330 | arginine and proline metabolism |  | 2.97E-07  12 |  |  |  |  | 8.09E-08  12 |
| KEGG5412 | arrhythmogenic right ventricular cardiomyopathy (ARVC) |  | 1.79E-06  13 |  |  |  |  | 4.04E-07  13 |
| KEGG53 | ascorbate and aldarate metabolism |  | 2.00E-04  6 |  |  |  |  | 1.10E-03  5 |
| KEGG4360 | axon guidance |  | 1.79E-06  17 |  |  |  |  | 2.35E-06  16 |
| KEGG4662 | B cell receptor signaling pathway |  | 2.73E-07  14 |  |  |  |  | 5.43E-07  13 |
| KEGG5217 | Basal cell carcinoma |  | 1.10E-02  6 |  |  |  |  | 7.14E-03  6 |
| KEGG3022 | Basal transcription factors |  | 7.70E-03  5 |  |  |  |  | 4.65E-03  5 |
| KEGG3410 | Base excision repair |  | 1.20E-03  6 |  |  |  |  | 6.58E-04  6 |
| KEGG5219 | bladder cancer |  | 1.51E-06  10 |  |  |  |  | 6.15E-07  10 |
| KEGG4020 | calcium signaling pathway |  | 4.47E-08  23 |  |  |  |  | 2.20E-08  22 |
| KEGG4260 | cardiac muscle contraction |  | 3.03E-12  20 |  |  |  |  | 2.18E-13  20 |
| KEGG4514 | cell adhesion molecules (CAMs) |  | 3.38E-08  20 |  |  |  |  | 2.81E-06  16 |
| KEGG4110 | cell cycle |  | 1.67E-06  17 |  |  |  |  | 2.61E-07  17 |
| KEGG4062 | chemokine signaling pathway |  | 2.32E-19  39 |  |  |  |  | 4.43E-21  39 |
| KEGG5220 | chronic myeloid leukemia |  | 7.00E-10  17 |  |  |  |  | 1.54E-10  17 |
| KEGG20 | citrate cycle (TCA cycle) |  | 1.17E-05  8 |  |  |  |  | 3.46E-06  **8** |
| KEGG5210 | colorectal cancer |  | 5.67E-10  18 |  |  |  |  | 1.19E-10  10 |
| KEGG4610 | complement and coagulation cascades |  | 2.13E-05  11 |  |  |  |  | 5.20E-05  10 |
| KEGG4060 | cytokine-cytokine receptor interaction |  | 8.04E-19  45 |  |  |  |  | 1.06E-20  45 |
| KEGG5414 | dilated cardiomyopathy |  | 4.84E-11  20 |  |  |  |  | 6.05E-12  20 |
| KEGG3030 | DNA replication |  | 1.40E-03  6 |  |  |  |  | 8.90E-04  6 |
| KEGG4320 | dorso-ventral axis formation |  | 1.91E-05  7 |  |  |  |  | 7.85E-06  7 |
| KEGG982 | drug metabolism - cytochrome P450 |  | 1.04E-06  13 |  |  |  |  | 9.78E-06  11 |
| KEGG983 | Drug metabolism - other enzymes |  | 1.90E-03  7 |  |  |  |  | 4.12E-03  6 |
| KEGG04512 | ECM-receptor interaction |  | 1.15E-15  24 |  | 1.20E-05  24 |  | 3.91E-02  24 | 1.51E-15  23 |
| KEGg4144 | endocytosis |  | 4.09E-13  31 |  |  |  |  | 1.70E-11  27 |
| KEGg5213 | endometrial cancer |  | 2.41E-08  13 |  |  |  |  | 7.60E-09  13 |
| KEGG5120 | epithelial cell signaling in Helicobacter pylori infection |  | 1.83E-13  20 |  | 6.30E-05  20 |  | 3.59E-02  20 | 2.27E-13  19 |
| KEGG4012 | ErbB signaling pathway |  | 4.67E-08  16 |  |  |  |  | 1.19E-08  16 |
| KEGG4664 | Fc epsilon RI signaling pathway |  | 7.93E-08  15 |  |  |  |  | 1.91E-08  15 |
| KEGG4666 | Fc gamma R-mediated phagocytosis |  | 1.31E-10  20 |  |  |  |  | 1.32E-11  20 |
| KEGG4510 | focal adhesion |  | 1.38E-27  49 |  | 0.00E+00  49 | 1.81E-07  48 | 1.63E-03  49 | 9.56E-29  48 |
| KEGG4540 | gap junction |  | 2.17E-06  14 |  |  |  |  | 3.13E-06  13 |
| KEGG5214 | glioma |  | 3.20E-07  13 |  |  |  |  | 7.43E-08  13 |
| KEGG480 | glutathione metabolism |  | 7.31E-06  10 |  |  |  |  | 3.13E-06  10 |
| KEGG561 | Glycerolipid metabolism |  | 4.30E-03  6 |  |  |  |  | 2.22E-03  6 |
| KEGG260 | glycine, serine and threonine metabolism |  | 7.00E-04  6 |  |  |  |  | 3.96E-04  6 |
| KEGG10 | glycolysis /gluconeogenesis |  | 1.31E-06  12 |  |  |  |  | 2.78E-06  11 |
| KEGG4912 | GnRH signaling pathway |  | 6.37E-08  17 |  |  |  |  | 1.01E-08  17 |
| KEGG4340 | hedgehog signaling pathway |  | 1.00E-04  9 |  |  |  |  | 5.53E-05  9 |
| KEGG4640 | hematopoietic cell lineage |  | 3.20E-07  15 |  |  |  |  | 7.95E-08  15 |
| KEGG340 | Histidine metabolism |  | 3.30E-03  5 |  |  |  |  | 2.15E-03  5 |
| KEGG5016 | Huntington's disease |  | 1.91E-21  41 |  | 2.30E-05  41 | 2.65E-06  41 |  | 8.09E-24  41 |
| KEGG5410 | hypertrophic cardiomyopathy (HCM) |  | 1.17E-11  20 |  |  |  |  | 1.23E-12  20 |
| KEGG4910 | insulin signaling pathway |  | 1.02E-08  21 |  |  |  |  | 1.28E-09  21 |
| KEGG4672 | intestinal immune network for IgA production |  | 4.00E-04  8 |  |  |  |  | 3.77E-03  6 |
| KEGG4630 | Jak-STAT signaling pathway |  | 1.37E-06  19 |  |  |  |  | 3.20E-07  19 |
| KEGg4670 | leukocyte transendothelial migration |  | 2.67E-12  24 |  |  |  |  | 1.27E-13  24 |
| KEGG4730 | long-term depression |  | 2.39E-05  11 |  |  |  |  | 8.68E-06  11 |
| KEGG4142 | lysosome |  | 4.11E-05  14 |  |  |  |  | 5.52E-05  13 |
| KEGG4010 | MAPK signaling pathway |  | 2.98E-14  39 |  |  |  |  | 1.31E-13  36 |
| KEGG4916 | melanogenesis |  | 7.00E-04  11 |  |  |  |  | 2.34E-04  11 |
| KEGG5218 | melanoma |  | 9.10E-07  13 |  |  |  |  | 2.59E-07  13 |
| KEGG980 | metabolism of xenobiotics by cytochrome P450 |  | 4.46E-06  12 |  |  |  |  | 5.20E-05  10 |
| KEGG3430 | mismatch repair |  | 1.00E-04  6 |  |  |  |  | 6.97E-05  6 |
| KEGG4150 | mTOR signaling pathway |  | 1.03E-05  10 |  |  |  |  | 4.26E-06  10 |
| KEGG4650 | natural killer cell mediated cytotoxicity |  | 2.55E-13  27 |  |  |  |  | 1.92E-11  23 |
| KEGG4080 | Neuroactive ligand-receptor interaction | 7.21E-06  14 | 4.21E-02  14 |  |  |  |  |  |
| KEGG4722 | neurotrophin signaling pathway |  | 1.04E-11  24 |  |  |  |  | 9.03E-13  24 |
| KEGG510 | N-Glycan biosynthesis |  | 4.80E-03  6 |  |  |  |  | 3.08E-03  6 |
| KEGG910 | Nitrogen metabolism |  | 7.90E-03  4 |  |  |  |  | 5.86E-03  4 |
| KEGG4621 | NOD-like receptor signaling pathway |  | 2.57E-08  14 |  |  |  |  | 7.47E-09  14 |
| KEGG5223 | non-small cell lung cancer |  | 4.12E-09  14 |  |  |  |  | 1.15E-09  14 |
| KEGG3420 | nucleotide excision repair |  | 7.00E-04  7 |  |  |  |  | 4.22E-04  7 |
| KEGG4114 | oocyte meiosis |  | 1.37E-08  19 |  |  |  |  | 8.01E-09  18 |
| KEGG190 | oxidative phosphorylation |  | 6.21E-19  33 |  | 2.40E-05  33 |  | 3.11E-02  32 | 3.85E-20  32 |
| KEGG4115 | p53 signaling pathway |  | 9.36E-08  14 |  |  |  |  | 2.34E-08  14 |
| KEGG5212 | pancreatic cancer |  | 3.23E-09  16 |  |  |  |  | 7.63E-10  16 |
| KEGG5012 | Parkinson's disease |  | 5.61E-20  34 |  | 5.00E-06  34 | 4.17E-07  34 |  | 2.80E-22  34 |
| KEGG5130 | pathogenic Escherichia coli infection |  | 7.94E-07  12 |  |  |  |  | 8.26E-07  11 |
| KEGG5200 | pathways in cancer |  | 1.97E-32  67 |  | 1.00E-06  67 | 1.02E-06  66 |  | 2.46E-35  67 |
| KEGG4070 | Phosphatidylinositol signaling system |  | 1.42E-02  7 |  |  |  |  | 7.64E-03  7 |
| KEGG860 | porphyrin and chlorophyll metabolism |  | 5.00E-04  7 |  |  |  |  | 1.77E-03  6 |
| KEGG3320 | PPAR signaling pathway |  | 5.00E-04  9 |  |  |  |  | 2.78E-04  9 |
| KEGG5340 | Primary immunodeficiency |  | 6.90E-03  5 |  |  |  |  | 4.65E-03  5 |
| KEGG5020 | prion diseases |  | 2.21E-05  8 |  |  |  |  | 8.01E-05  7 |
| KEGG4914 | progesterone-mediated oocyte maturation |  | 1.88E-12  21 |  |  |  |  | 2.18E-13  21 |
| KEGG5215 | prostate cancer |  | 4.09E-13  22 |  |  |  |  | 6.79E-14  22 |
| KEGG3050 | proteasome |  | 9.25E-17  20 | 7.17E-03  20 | 0.00E+00  20 |  |  | 4.42E-17  19 |
| KEGG3060 | Protein export |  | 3.70E-03  3 |  |  |  |  | 6.97E-05  6 |
| KEGG230 | purine metabolism |  | 9.56E-07  19 |  |  |  |  | 3.48E-07  19 |
| KEGG240 | pyrimidine metabolism |  | 2.43E-07  16 |  |  |  |  | 6.37E-08  16 |
| KEGG620 | pyruvate metabolism |  | 8.00E-06  9 |  |  |  |  | 3.55E-06  9 |
| KEGG4810 | regulation of actin cytoskeleton |  | 3.23E-12  32 |  |  |  |  | 1.25E-13  32 |
| KEGG5211 | renal cell carcinoma |  | 2.36E-10  17 |  |  |  |  | 5.17E-11  17 |
| KEGG3010 | ribosome |  | 2.32E-19  28 |  | 4.80E-05  26 |  |  | 1.04E-20  28 |
| KEGG4622 | RIG-I-like receptor signaling pathway |  | 5.12E-06  12 |  |  |  |  | 2.00E-06  12 |
| KEGG3018 | RNA degradation |  | 3.90E-03  7 |  |  |  |  | 3.82E-04  8 |
| KEGG5222 | small cell lung cancer |  | 1.30E-14  23 |  | 5.80E-05  23 |  | 4.92E-02  23 | 1.51E-15  23 |
| KEGG3040 | spliceosome |  | 5.09E-10  22 |  |  |  |  | 2.70E-10  21 |
| KEGG4660 | T cell receptor signaling pathway |  | 1.31E-10  21 |  |  |  |  | 1.52E-10  20 |
| KEGG4742 | Taste transduction |  | 3.06E-02  5 |  |  |  |  | 4.12E-03  6 |
| KEGG4350 | TGF-beta signaling pathway |  | 1.33E-10  19 |  |  |  |  | 2.05E-11  19 |
| KEGG5216 | thyroid cancer |  | 5.17E-05  7 |  |  |  |  | 2.97E-05  7 |
| KEGG4530 | tight junction |  | 1.21E-05  16 |  |  |  |  | 2.57E-06  16 |
| KEGG4620 | toll-like receptor signaling pathway |  | 3.77E-11  21 |  |  |  |  | 6.22E-12  21 |
| KEGG380 | Tryptophan metabolism |  | 1.17E-02  5 |  |  |  |  | 7.31E-03  5 |
| KEGG4930 | Type II diabetes mellitus |  | 5.30E-03  6 |  |  |  |  | 3.41E-03  6 |
| KEGG4120 | Ubiquitin mediated proteolysis |  | 1.69E-02  10 |  |  |  |  | 7.67E-03  10 |
| KEGG4270 | Vascular smooth muscle contraction |  | 1.90E-03  11 |  |  |  |  | 6.54E-04  11 |
| KEGG4370 | VEGF signaling pathway |  | 1.79E-06  13 |  |  |  |  | 3.30E-06  12 |
| KEGG5110 | vibrio cholerae infection |  | 5.57E-08  13 |  |  |  |  | 8.09E-08  12 |
| KEGG5416 | viral myocarditis |  | 2.90E-08  15 |  |  |  |  | 9.78E-06  11 |
| KEGG4310 | Wnt signaling pathway |  | 5.01E-05  16 |  |  |  |  | 5.51E-05  15 |
| Number of significant categories only with this tool | | 0 | 22 | 0 | 0 | 0 | 0 | 2 |
