# Supplementary material for: Consensus Pathways Implicated in Prognosis of Colorectal Cancer Identified Through Systematic Enrichment Analysis of Gene Expression Profiling Studies
Source: PLoS One. 2011 Apr 25;6(4):e18867. doi: 10.1371/journal.pone.0018867 (PMC3081819; doi:10.1371/journal.pone.0018867)
Supplement: Table S6 — Number of overrepresented GO and KEGG categories reported by more than one enrichment tool. (DOC) [file pone.0018867.s008.doc]

**Table S6**. Number of overrepresented GO and KEGG categories reported by more than one enrichment tool.

|  | **54 gene list** | | | **124 gene list** | | | **1475 gene list** | | |
| --- | --- | --- | --- | --- | --- | --- | --- | --- | --- |
|  | **BP** | **MF** | **KEGG** | **BP** | **MF** | **KEGG** | **BP** | **MF** | **KEGG** |
| Significant categories ≥ 2 tools | 28 | 9 | **8** | 30 | 22 | **17** | 206 | 40 | 114 |
| Significant categories ≥ 3 tools | **15** | **5** | 2 | **16** | **17** | 2 | 102 | 25 | 10 |
| Significant categories ≥ 4 tools | 3 | 1 | 0 | 4 | 4 | 1 | 37 | 14 | **9** |
| Significant categories ≥ 5 tools | 2 | 0 | 0 | 0 | 1 | 1 | **14** | **9** | 1 |

BP, Gene Ontology Biological Process; MF, Gene Ontology Molecular Function; KEGG, Kyoto Encyclopedia of Genes and Genomes. A threshold of at least five common enriched categories (indicated in bold) was used to select the consistently enriched categories.
