# Supplementary material for: Consensus Pathways Implicated in Prognosis of Colorectal Cancer Identified Through Systematic Enrichment Analysis of Gene Expression Profiling Studies
Source: PLoS One. 2011 Apr 25;6(4):e18867. doi: 10.1371/journal.pone.0018867 (PMC3081819; doi:10.1371/journal.pone.0018867)
Supplement: Table S7 — Result of the enrichment analysis in four individual GEP studies for the consistently enriched GO and KEGG categories of the global analysis. (DOC) [file pone.0018867.s009.doc]

**Table S7**. Result of the enrichment analysis in four individual GEP studies for the consistently enriched GO and KEGG categories of the global analysis.

| **ID** | **Category** | **Group of categories with biological significance** | **Arango et al.** | **Bertucci et al.** | **Fritzmann et al.** | **Jorissen et al.** |
| --- | --- | --- | --- | --- | --- | --- |
| **GO Biological Process (8 tools)** | |  |  |  |  |  |
| **GO:0008283** | **cell proliferation** | a) | 0 | 3 tools | 1 tool | 0 |
| **GO:0048518** | **positive regulation of biological process** | a) | 0 | 3 tools | 0 | 0 |
| **GO:0048522** | **positive regulation of cellular process** | a) | 0 | 3 tools | 0 | 0 |
| **GO:0042981** | **regulation of apoptosis** | a) | 4 tools | 4 tools | 0 | 0 |
| **GO:0042127** | **regulation of cell proliferation** | a) | 0 | 1 tool | 0 | 0 |
| **GO:0042221** | **response to chemical stimulus** | a) | 2 tools | 3 tools | 0 | 3 tools |
| **GO Molecular Function (7 tools)** | |  |  |  |  |  |
| **GO:0015078** | **hydrogen ion transmembrane transporter activity** | b) | 0 | 5 tools | 2 tools | 0 |
| **GO:0022890** | **inorganic cation transmembrane transporter activity** | b) | 0 | 3 tools | 0 | 0 |
| **GO:0015077** | **monovalent inorganic cation transmembrane transporter activity** | b) | 0 | 5 tools | 2 tools | 0 |
| **GO:0005515** | **protein binding** | a) c) | 5 tools | 4 tools | 4 tools | 5 tools |
| **GO:0051082** | **unfolded protein binding** | a) | 7 tools | 1 tool | 3 tools | 0 |
| **KEGG pathway (7 tools)** | |  |  |  |  |  |
| **KEGG4512** | **ECM-receptor interaction** | c) | 0 | 3 tools | 2 tools | 7 tools |
| **KEGG4510** | **focal adhesion** | c) | 2 tools | 3 tools | 0 | 7 tools |
| **KEGG5016** | **Huntington's disease** | b) | 4 tools | 6 tools | 3 tools | 0 |
| **KEGG190** | **oxidative phosphorylation** | b) | 1 tool | 7 tools | 3 tools | 0 |
| **KEGG5200** | **pathways in cancer** | a) | 3 tools | 3 tools | 2 tools | 0 |
| **KEGG5012** | **Parkinson's disease** | b) | 1 tool | 6 tools | 3 tools | 0 |
| **KEGG5222** | **small cell lung cancer** |  | 3 tools | 3 tools | 0 | 2 tools |

a) Categories related to cell proliferation, apoptosis and protein binding; b) Oxidative phosphorylation and related categories; c) Extracellular matrix receptor interaction and related categories.
