# Supplementary material for: Consensus Pathways Implicated in Prognosis of Colorectal Cancer Identified Through Systematic Enrichment Analysis of Gene Expression Profiling Studies
Source: PLoS One. 2011 Apr 25;6(4):e18867. doi: 10.1371/journal.pone.0018867 (PMC3081819; doi:10.1371/journal.pone.0018867)
Supplement: Table S8 — Enrichment tools used and their characteristics. (DOC) [file pone.0018867.s010.doc]

**Table S8**. Enrichment tools used and their characteristics.

| **Tool name** | **First reference** | **Databases** | **Key statistical method** | **Multiple testing correction method(s)** |
| --- | --- | --- | --- | --- |
| ConsensusPathDB | [1] | KEGG | Hypergeometric | FDR |
| DAVID | [2] | BP / MF / KEGG | EASE score (Fisher‘s exact) | Benjamini* / FDR / Bonferroni |
| FatiGO | [3] | BP / MF | Fisher‘s exact | 3 methods (including B-H) |
| GATHER | [4] | BP / KEGG | Bayes factor | FDR |
| GeneCodis | [5] | BP / MF / KEGG | Hypergeometric | FDR |
| GOTM | [6] | BP / MF | Hypergeometric | B-H |
| g:Profiler | [7] | BP / MF / KEGG | Hypergeometric | g:SCS threshold |
| Ingenuity^#^ | Trial licence commercial software | own databases | Fisher‘s exact | B-H |
| ToppFun | [8] | BP / MF / KEGG | Hypergeometric | Bonferroni / FDR* |
| WebGestalt | [9] | BP / MF / KEGG | Hypergeometric | B-H |

BP, Gene Ontology Biological Process; MF, Gene Ontology Molecular Function; KEGG, Kyoto Encyclopedia of Genes and Genomes; FDR, false discovery rate; B-H, Benjamini-Hochberg. *Indicates the multiple testing correction method used, if more than one method possible. #The Ingenuity software makes use of its own databases for enrichment analyses, not the freely available Gene Ontology and KEGG. Both Top Bio Functions (equivalent to GO categories) and Top Canonical Pathways (equivalent to KEGG pathways) tools included in the Ingenuity software were used.

**REFERENCES**

1. Kamburov A, Wierling C, Lehrach H, Herwig R (2009) ConsensusPathDB--a database for integrating human functional interaction networks. Nucleic Acids Res 37: D623-628.

2. Huang da W, Sherman BT, Lempicki RA (2009) Systematic and integrative analysis of large gene lists using DAVID bioinformatics resources. Nat Protoc 4: 44-57.

3. Al-Shahrour F, Diaz-Uriarte R, Dopazo J (2004) FatiGO: a web tool for finding significant associations of Gene Ontology terms with groups of genes. Bioinformatics 20: 578-580.

4. Chang JT, Nevins JR (2006) GATHER: a systems approach to interpreting genomic signatures. Bioinformatics 22: 2926-2933.

5. Carmona-Saez P, Chagoyen M, Tirado F, Carazo JM, Pascual-Montano A (2007) GENECODIS: a web-based tool for finding significant concurrent annotations in gene lists. Genome Biol 8: R3.

6. Zhang B, Schmoyer D, Kirov S, Snoddy J (2004) GOTree Machine (GOTM): a web-based platform for interpreting sets of interesting genes using Gene Ontology hierarchies. BMC Bioinformatics 5: 16.

7. Reimand J, Kull M, Peterson H, Hansen J, Vilo J (2007) g:Profiler--a web-based toolset for functional profiling of gene lists from large-scale experiments. Nucleic Acids Res 35: W193-200.

8. Chen J, Bardes EE, Aronow BJ, Jegga AG (2009) ToppGene Suite for gene list enrichment analysis and candidate gene prioritization. Nucleic Acids Res 37: W305-311.

9. Zhang B, Kirov S, Snoddy J (2005) WebGestalt: an integrated system for exploring gene sets in various biological contexts. Nucleic Acids Res 33: W741-748.
